# The Vacuolar Inositol Transporter *Bv*INT1;1 Contributes to Raffinose Biosynthesis and Reactive Oxygen Species Scavenging During Cold Stress in Sugar Beet

**DOI:** 10.1111/pce.15367

**Published:** 2025-01-08

**Authors:** Johannes Berg, Cristina Martins Rodrigues, Claire Scheid, Yana Pirrotte, Cristiana Picco, Joachim Scholz‐Starke, Wolfgang Zierer, Olaf Czarnecki, Dieter Hackenberg, Frank Ludewig, Wolfgang Koch, H. Ekkehard Neuhaus, Christina Müdsam, Benjamin Pommerrenig, Isabel Keller

**Affiliations:** ^1^ University of Kaiserslautern Plant Physiology, Paul‐Ehrlich‐Str. Kaiserslautern Germany; ^2^ Istituto di Biofisica Consiglio Nazionale delle Ricerche (CNR) Via De Marini Genova Italy; ^3^ Friedrich‐AlexanderUniversity of Erlangen‐Nuremberg Biochemistry, Staudtstr Erlangen Germany; ^4^ KWS SAAT SE & Co. KGaA Grimsehlstr. Einbeck Germany

**Keywords:** cold stress, inositol, raffinose family oligosaccharides (RFO), reactive oxygen species (ROS), sugar beet, transport protein, vacuole

## Abstract

Despite a high sucrose accumulation in its taproot vacuoles, sugar beet (*Beta vulgaris* subsp. *vulgaris*) is sensitive to freezing. Earlier, a taproot‐specific accumulation of raffinose was shown to have beneficial effects on the freezing tolerance of the plant. However, synthesis of raffinose and other oligosaccharides of the raffinose family depends on the availability of *myo*‐inositol. Since inositol and inositol‐metabolising enzymes reside in different organelles, functional inositol metabolism and raffinose synthesis depend on inositol transporters. We identified five homologues of putative inositol transporters in the sugar beet genome, two of which, *Bv*INT1;1 and *Bv*INT1;2, are localised at the tonoplast. Among these, only the transcript of *BvINT1;1* is highly upregulated in sugar beet taproots under cold. *Bv*INT1;1 exhibits a high transport specificity for inositol and sugar beet mutants lacking functional *Bv*INT1;1 contain increased inositol levels, likely accumulating in the vacuole, and decreased raffinose contents under cold treatment. Due to the quenching capacity of raffinose for Reactive Oxygen Species (ROS), which accumulate under cold stress, *bvint1;1* sugar beet plants show increased expression of both, ROS marker genes and detoxifying enzymes. Based on these findings, we conclude that the vacuolar inositol transporter *Bv*INT1;1 is contributing to ROS‐homoeostasis in the cold metabolism of sugar beet.

## Summary statement

- Inositol exported from the vacuole via the tonoplast inositol transporter *Bv*INT1;1 contributes to raffinose synthesis and scavenging of reactive oxygen species in sugar beet (*Beta vulgaris* subsp. *vulgaris*) taproots during cold.

## 1 Introduction

Stress is defined as any condition affecting plant metabolism, development, or growth, potentially impairing their reproduction (Lichtenthaler 1998; Rihan, Al‐Issawi, and Fuller 2017). Roughly 64% of the Earth's land surface exhibits an average minimum temperature below 0°C (Rihan, Al‐Issawi, and Fuller 2017). Air temperatures can easily fluctuate by 15°C or more between day and night (Parton and Logan 1981). Thus, environmental temperature, mainly low temperatures, represents one of the most critical abiotic stressors that plants encounter during their lifecycle. While temperatures slightly above 0°C are already low enough to cause reduced growth, mainly because of slowed enzymatic reactions and stiffening of membranes (Yadav 2009), freezing can result in the formation of intra‐ and extracellular ice crystals, leading to the rupture of cell walls and plasma membranes, or loss of cellular water (Burke et al. 1976; Wolfe and Bryant 1999).

To avoid such damage and to increase freezing tolerance, many plants respond to chilling (0°C–4°C) with a molecular reorganisation named ‘cold acclimation’ (Yadav 2009; Fürtauer et al. 2019). At the physiological level, protective compounds such as soluble sugars, amino acids, organic acids, sugar alcohols, or cold tolerance proteins are synthesised and accumulate in plant cells during acclimation to low temperatures (Kaplan et al. 2007; Pommerrenig et al. 2018; Ding, Shi, and Yang 2019). These compounds are also involved in regulating the osmotic potential, reducing ice crystal formation, and stabilising enzymes, membranes, and other cellular components (Yadav 2009; Guy 1990). In addition, some of the accumulating substances especially sugars together with antioxidants, can scavenge Reactive Oxygen Species (ROS), the formation of which is generally stimulated under stress conditions (Suzuki and Mittler 2006; Korn et al. 2008; Keunen et al. 2013).

The production of ROS is not intrinsically harmful to the plant as ROS are also involved in important feedback and signalling responses. However, oxidative stress and damage can occur if the homoeostasis between ROS production and scavenging is imbalanced (Gill and Tuteja 2010). Chloroplasts are the major source of ROS in autotrophic tissues. There, ROS formation is favoured when the absorbed light energy exceeds the photosynthetic capacity, leading to excessive reduction of the photosynthetic electron transport chain and subsequent electron transfer to alternative acceptors such as oxygen (Sewelam, Kazan, and Schenk 2016). While chloroplasts are the main source of ROS in photosynthetic tissues, mitochondria are the major site of ROS production in heterotrophic tissues. There, electrons from the mitochondrial electron transport chain can be transferred to oxygen mainly at Complexes I to III (Choudhury et al. 2017; Huang et al. 2016). ROS production increases sharply when enzymatic reactions, such as those in the Calvin–Benson–Bassham Cycle (CBB) or the mitochondrial respiration are slowed down, as is the case at low temperatures.

The cellular consequences caused by an imbalance in ROS homoeostasis can be detrimental. Aberrant ROS can cause damage to proteins, membranes, DNA and RNA (Decros et al. 2019; Baek and Skinner 2012). A significant impact of ROS‐induced oxidative damage can be observed in sugar beet (*Beta vulgaris* subsp. *vulgaris*) under low‐temperature stress (Keller, Müdsam, et al. 2021). There, the pith tissue in the neck of the taproot shows a particularly high accumulation of ROS accompanied by a high variance in its sensitivity to freezing. Sugar beet genotypes with a low accumulation of the trisaccharide raffinose in their pith exhibit cellular damage at low temperatures, indicating a high level of protection of this metabolite against the increased ROS imbalance (Keller, Müdsam, et al. 2021).

In general, raffinose or other carbohydrates of the raffinose family oligosaccharide (RFO) family, such as stachyose, are known to accumulate in various plant species when exposed to abiotic stress (Taji et al. 2002; Saito and Yoshida 2011; Li et al. 2020). Raffinose is synthesised by the transfer of galactosyl units from a galactinol moiety to sucrose. Its synthesis involves the enzymes galactinol synthase (GOLS) and raffinose synthase (RS), whose activities are known to be strongly upregulated by chilling (Cunningham et al. 2003; Egert, Keller, and Peters 2013; Sprenger and Keller 2000; Zuther et al. 2004). However, the entire metabolic process of RFO synthesis depends on the availability of the polyol inositol in the cytosol, since inositol, together with UDP‐galactose, is one of the two substrates required for the synthesis of galactinol, the galactosyl donor for RFO synthesis (Keller, Rodrigues, et al. 2021).

Inositol is not only important for RFO synthesis but also plays important roles in cellular phosphate storage (Loewus and Loewus 1983), cell wall and membrane biosynthesis (Loewus and Murthy 2000), intra‐ and intercellular communication (Cote and Crain 1993; Munnik, Irvine, and Musgrave 1998; Perera, Heilmann, and Boss 1999), and the storage and transport of plant hormones such as auxin (Cohen and Bandurski 1978, Kowalczyk and Bandurski 1991). Because of their diverse functions, enzymes involved in cellular inositol metabolism are found in almost every organelle of the plant cell. Accordingly, functional inositol metabolism relies on transport proteins that mediate the efficient transfer of inositol and inositol intermediates between organelles.

In Arabidopsis, four transport proteins with high inositol specificity (*At*INT1 to *At*INT4) mediate inositol transport across the plasma membrane (*At*INT2 and *At*INT4; Schneider et al. 2007; Schneider et al. 2006) or the tonoplast into the cytosol (*At*INT1; Schneider et al. 2008). In this context, the tonoplast‐located protein *At*INT1 appears to be critical for the full functionality of plant metabolism, as *atint1;1* knockout mutants show increased inositol levels and impaired growth under standard conditions (Schneider et al. 2008).

However, findings on sugar beet and other plant species like rice (*Oryza sativa*), maize (*Zea mays*), or *Medicago falcata* demonstrated increased inositol metabolism and especially RFO accumulation under stress conditions (Zhuo et al. 2013: Yang et al. 2019, Guo et al. 2021; Keller, Müdsam, et al. 2021). Given the different cellular compartmentalisation of inositol pools and of raffinose biosynthesis enzymes, cold‐dependent inositol transport processes must be postulated. We, therefore, investigated such processes in sugar beet (*Beta vulgaris* subsp. *vulgaris*), which accumulates inositol‐derived RFOs in its pith tissue under cold stress. Thus, we quantified the cold‐dependent expression of inositol transporters, their subcellular localisation, transport properties, and their impact on the cold metabolism of the plant.

## 2 Materials and Methods

### 2.1 Generation of Constructs for Transient Transformation of Arabidopsis Protoplasts and Xenopus Oocytes

The coding sequence (CDS) of sugar beet inositol transporter homologues were amplified from *Beta vulgaris* subsp. *vulgaris* cDNA using the primers *Bv*INT1;1–5′BspHI‐f and *Bv*INT1;1–3′BspHI‐r (for *BvINT1;1*; Refbeet 1.2.2 transcript ID Bv3_054860_wjth.t2), *Bv*INT1;2–5′NcoICC‐f and *Bv*INT1;2–3′ATGG‐r (for *BvINT1;2*; Refbeet 1.2.2 transcript ID Bv3_054850_pyaq.t1), or *Bv*INT2–5′TCATG‐f and *Bv*INT2–3′GBspHI‐r (for *BvINT2*; Refbeet 1.2.2 transcript ID Bv8_184790_ygxh.t1), removing the stop‐codon and introducing flanking *BspHI‐* (*BvINT1;1* and *BvINT2*), or *NcoI‐* (*BvINT1;2*) restriction sites (Supporting Information S2: Table S1). PCR fragments were sub‐cloned into pCRblunt (Life Technologies, Carlsbad, US), and correct insert sequences were verified by sequencing. To generate fluorophore fusion constructs, expressed under the control of the *35S* promoter for transient transformation of mesophyll protoplasts, INT (Inositol Transporter) coding fragments were excised from pCRblunt with *Nco*I or *BspHI* as indicated above and inserted into the unique *Nco*I cloning sites of pCS120 (Dotzauer et al. 2010), pSS87 (Schneider et al. 2012), and pSE35e‐C (Müdsam et al. 2018) yielding fusions of Green Fluorescent Protein (GFP) (pCS120, pSS87) or Red Fluorescent Protein (RFP) (pSE35e‐C) to the C‐ (pCS120, pSE35e‐C) or N‐terminus (pSS87) of the putative transporters, respectively.

To introduce mutations in the CDS corresponding to the *Bv*INT1;1 C‐terminus (*Bv*INT1;1^LL489/490AA^) the forward primer *Bv*INT1;1‐5′BspHI‐f (Supporting Information S2: Table 1) was combined with INT1;1‐LL/AA‐X‐r (Supporting Information S2: Table S1) in a PCR using *Bv*INT1;1CDS/pCRblunt as a template to obtain fragments comprising modified *BvINT1;1* CDS up to an internal *Bsp119I*‐site within the *BvINT1;1* CDS. Each fragment was cloned in pCRblunt and verified by sequencing. Fragments comprising the modified CDS were excised from pCRblunt using *NcoI* (which cuts the *BvINT1;1* CDS 5′ of the introduced modification) and *Bsp119I* (which cuts the *BvINT1;1* CDS 3′ of the introduced modification) to replace the corresponding fragment of the native *BvINT1;1* CDS in the respective expression vectors. Isolated clones were partially sequenced (spanning the affected nucleotide range) to confirm the exchange of the native *BvINT1;1* for the respective modified fragment in the final expression vectors.

‘InFusion’ cloning was used to generate constructs for expression in Xenopus oocytes: *BvINT1;1* or *BvINT1;1* ^*LL489/490AA*^ CDS fragments were amplified from corresponding GFP‐fusion constructs (pCS120‐derivatives) using primer combinations (i) pNBI16/22‐BvINT1;1‐f and *Bv*INT1;1‐pNBI16/21‐r, (ii) pNBI21‐*Bv*INT1;1‐f and *Bv*INT1;1‐pNBI16/21‐r, or (iii) pNBI16/22‐*Bv*lNT1;1‐f and *Bv*INT1;1‐pNBI22‐r (Supporting Information S2: Table S1) to generate fragments suited for cloning into linearised (i) pNBI16, (ii) pNBI21, or (iii) pNBI22, which ultimately yielded unlabelled *Bv*INT1;1 or *Bv*INT1;1^LL489/490AA^ (pNBI16) or fusions of YFP to the N‐ (pNBI21) or C‐terminus (pNBI22) of the putative transporter.

### 2.2 Arabidopsis Protoplast Isolation, Polyethylene Glycol‐Mediated Transformation of Protoplasts

For transient transformation, Arabidopsis mesophyll protoplasts (Col‐0) were isolated (Drechsel et al. 2011) and transformed (Abel and Theologis 1994) as described. Transformed Arabidopsis protoplasts were incubated in the dark at 22°C for 48 h before confocal analysis. Protoplasts were lysed with mild osmotic shock to release vacuoles (Costa et al. 2012).

### 2.3 Confocal Microscopy

Images of protoplasts were captured on a confocal laser scanning microscope (Leica TCS SP5; Leica Microsystems) and processed with LAS AF Version 2.7.29586. Wavelengths of 488 and 561 nm (RFP) omitted by an Argon‐laser were used for the excitation of GFP or RFP, respectively. The detection window for GFP ranged from 495 to 553 nm for GFP when only GFP constructs had been transformed, or from 495 to 548 nm when RFP‐fusions had been co‐transformed. The detection window for RFP ranged from 591 to 640 nm. At least five independent images were analyzed per colocalization study.

### 2.4 Oocyte Expression and Two‐Electrode Voltage‐Clamp Recordings

Oocyte preparation, injection, and two‐electrode voltage‐clamp recordings were performed as described earlier (Picco et al. 2014, 2015). In vitro transcription of INT1,1^LL489/490AA^ cRNA was done using the mMESSAGE mMACHINE T7 kit (Life Technologies, Italy), and the integrity of the transcribed cRNA was confirmed by agarose gel analysis. About 25–50 ng cRNA per oocyte were injected. Whole‐cell membrane currents were recorded at room temperature and a holding potential of −20 mV, using a homemade two‐electrode voltage‐clamp amplifier. The standard bath solution contained (in mM): 96 NaCl, 2 KCl, 1 CaCl_2_, 1 MgCl_2_, 10 MES, adjusted to pH 5.5. In experiments performed at pH 7.5 or pH 8.5, MES was replaced by equimolar HEPES or Tris, respectively. For dose–response analyses, current amplitudes recorded from individual oocytes were plotted against the applied inositol concentration and subjected to a fit with the Michaelis–Menten equation: *I* = *I*_max_ × *c*/(*K*_m_ + *c*), where *I*_max_ is the maximum value at saturating inositol concentrations, *c* the inositol concentration and *K*_m_ the concentration at half‐maximal current.

### 2.5 Sugar Beet Plant Growth and Cold Treatment

Mutant plants, carrying a premature STOP codon in the *BvINT1;1* CDS (*Bv*INT1;1_W188STOP), further referred to as *bvint1;1*, were identified in an 0,5% EMS‐treated multigerm sugar beet population (Keygene, Wageningen, Netherlands) using a specific marker for the G to A SNP at position RefBeet‐1.2: Bvchr3.sca003:c4242591. The corresponding mutant was back‐crossed and subsequently self‐crossed to generate homozygous, heterozygous, and WT allele‐carrying segregants for subsequent analysis. Sugar beet plants were grown in pots (18 × 18 × 25cm) on standard soil substrate ED73 (Einheitserdwerke Patzer, Sinntal‐Altenngronau, Germany) with 10% (*v*/*v*) sand under a 10 h light/14 h dark cycle in the greenhouse with light intensities of 320 µmol m^−2^ s^−1^. Plants were grown for 12 weeks at 20°C. Afterward, the population was split into two halves, one remained at 20°C for five further weeks to serve as a 20°C control group (Supporting Information S1: Figure S1). The other half was transferred to 12°C and 4°C for 1 week each afterward. After the 4°C treatment, the temperature was lowered to 1°C for further 3 weeks (Supporting Information S1: Figure S1). After a total growth of 17 weeks, plants were harvested at 20°C and 1°C in four biological replicates for determination of biomass, metabolite contents, and gene expression analysis (Supporting Information S1: Figure S1 green line). Plant tissue was generally harvested during mid‐day, 4 h after the onset of light. For metabolite and RNA extraction, plant material was frozen in liquid nitrogen and pulverised using a Retsch MM301 mill (Retsch, Haan, Germany). For the determination of photosynthetic activity, a kinetics analysis was performed. Starting with sugar beet plants grown for 12 weeks at 20°C, chlorophyll fluorescence was measured weekly during transfer from 20°C to 12°C, 4°C and 1°C (Supporting Information S1: Figure S1 black line). To examine recovery from cold exposure, chlorophyll autofluorescence was further measured in plants retransferred to 20°C following treatment at 1°C.

### 2.6 Isolation of Vacuoles From Sugar Beet Taproot Tissue

Vacuoles were isolated from taproots of 17‐week‐old sugar beet plants, grown under standard conditions (Jung et al. 2015). Briefly, three taproots of each genotype were used for individual vacuole isolation and were harvested, thinly sliced, and chopped using razor blades. Chopped taproot samples were incubated in an ice‐cold collection medium (750 mM mannitol, 5 mM EDTA, 1 mM DTT, 50 mM Tris‐HCl, pH 7.6) for 30 min with slight agitation. Taproot material was filtered from the solution using a stainless‐steel sieve (100 µm mesh width) and sedimented by centrifugation (2000*g*, 10 min, 4°C). Remaining pellets were resuspended in collection buffer containing 30% Nycodenz (Axis‐Shield GmbH, Heidelberg, Germany), a self‐forming gradient. After centrifugation (1000*g*, 15 min, 4°C), vacuoles floated to the upper phase of the gradient. α‐mannosidase activity was determined in isolated vacuole samples as well as in crude extract after sonification (Boller and Kende 1979).

### 2.7 Chlorophyll Fluorescence Measurements

Photosynthetic activity was measured using an M‐Series system MINI‐IMAGING PAM fluorometer (Heinz Walz, Effeltrich, Germany) according to Keller, Müdsam, et al. 2021. Before measurement, three individual plants were darkened for 8 min to deplete photosystem II (PSII) energy. Chlorophyll fluorescence was measured upon repeated light pulses with photosynthetic active radiation (PAR) of 76 µmol photons m^−2^ s^−1^ every 20 s until saturation occurred. The measured fluorescence parameters (Fm, Fo, Fm′, Fo′) were used to calculate the effective quantum yield of photosynthesis [Y(II)] as well as the quantum yield of regulated non‐photochemical energy loss [Y(NPQ)] and non‐regulated non‐photochemical energy loss [Y(NO)]. Calculations were performed by the software ImagingWinGigE (Heinz Walz, Effeltrich, Germany).

### 2.8 Metabolite Extraction and Quantification

Soluble metabolites were extracted with 80% EtOH at 80°C for 1 h twice as described in Keller, Müdsam, et al. 2021. Therefore, 50 mg of frozen and pulverised sugar beet material were weighed in 80% EtOH before extraction. Combined extracts were vaporised in a vacuum concentrator (Eppendorf, Hamburg, Germany). Evaporated pellets were resolved in 1 mL _dd_H_2_O and pellets remaining from sugar extraction were washed with 500 µL 80% EtOH and 1 mL _dd_H_2_O for starch isolation. 200 µL _dd_H_2_O were added to the washed pellet and samples were autoclaved for 40 min at 121°C. 200 µL enzyme mix (5 U α‐Amylase; 5 U Amyloglucosidase; 200 mM Sodium‐Acetate; pH 4.8) was added to the autoclaved pellet and incubated at 37°C for 4 h for hydrolytic cleavage of starch.

Sugars (glucose, fructose, sucrose) and hydrolysed starch concentrations were measured using a NAD^+^‐coupled enzymatic (Stitt et al. 1989), while sugar alcohols, raffinose, and galactinol contents were quantified via chromatography (Ho et al. 2020).

### 2.9 RNA Isolation and cDNA Synthesis

RNA was isolated from 50 mg plant material using the NucleoSpin RNA Plant Kit (Machery‐Nagel, Düren, Germany) according to the manufacturer's instructions. The final elution of RNA was performed in 30 µL RNAse‐free _dd_H_2_O.

One microgram of isolated RNA was reverse‐transcribed using the qScript cDNA synthesis kit (Quanta Biosciences, Beverly, MA, USA) according to the associated protocol.

### 2.10 Expression Analysis via RT‐qPCR

Gene expression analysis in sugar beet leaves and taproots at 20°C and 1°C was performed by reverse transcription quantitative PCR (RT‐qPCR) using the primers listed in Supporting Information S2: Table S1. Expression was analyzed in four biological replicates for each tested gene under each test condition. Relative expression was calculated via the Δ*C*_T_ method relative to the expression of *BvUBC9* (BVRB_1g003580; Supporting Information S2: Table S1).

### 2.11 RNAseq Data Extraction

Expression data of sugar beet INT‐transporters in leaves and taproots of sugar beet plants grown at 20°C, 4°C and 0°C were extracted from BioProject PRJNA602804, which was published in Rodrigues et al. 2020.

### 2.12 Phylogeny

Genetic distances of INT‐transporters in *Arabidopsis thaliana* and *Beta vulgaris* was calculated using the www.phylogeny.fr ‘one‐click’ mode (Dereeper et al. 2008). Phylogenetic data obtained via Phylogeny.fr was loaded into FigTree v1.4.4 for graphical representation.

### 2.13 Statistical Analysis

The student's *t*‐test was calculated using Microsoft Excel. One‐way ANOVA with posthoc Tukey HSD Test results were determined using the Calculator from Navendu Vasavada (https://astatsa.com/OneWay_Anova_with_TukeyHSD), while two‐way ANOVA was calculated using the two‐way ANOVA with posthoc Tukey HSD Test Calculator from Houssein Assaad (https://houssein‐assaad.shinyapps.io/TwoWayANOVA/; Assaad et al. 2015).

### 2.14 Phyre^2^ Modelling

For the prediction of functional and truncated *Bv*INT1;1 structure the ‘normal mode’ of Phyre^2^ (Kelley et al. 2015) was queried using the corresponding amino acid sequence as input.

## 3 Results

### 3.1 *BvINT1;1* Is Induced by Low Temperature in Taproots But Not in Shoots

**Figure 1:**
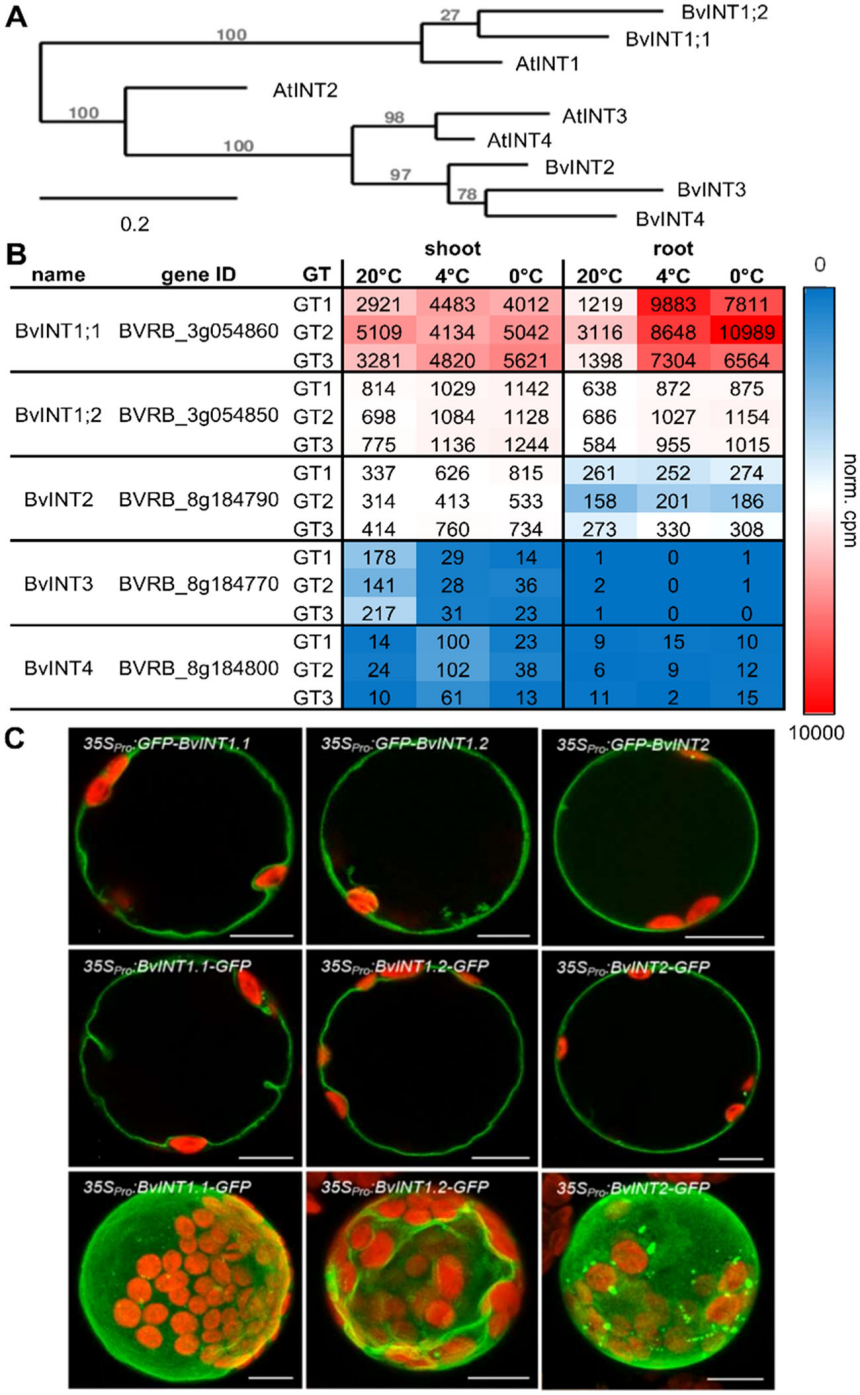
Characteristics of INT‐proteins in *Beta vulgaris*. (A) Phylogenetic tree of INT proteins from *Arabidopsis thaliana* and *B. vulgaris*. Predicted (*At*INT3: At2g35740) and confirmed (*At*INT1: At2g43330; *At*INT2: At1g30220; *At*INT4: At4g16480) amino acid sequences of the four Arabidopsis *At*INTs and five homologous proteins from *B. vulgaris* were aligned and a tree was calculated using *phylogeny.fr* one‐click mode. Sugar beet INTs were named according to their closest *A. thaliana* homologue, with *Bv*INT1;1: BVRB_3F054860.; *Bv*INT1;2: BVRB_3F054850; *Bv*INT1: BVRB_8g184790; *Bv*INT3: BVRB_8g184770; *Bv*INT4: BVRB_8g184800. (B) Heatmap representation of INT gene expression in sugar beet leaf‐ and taproot tissue at 20°C and under cold treatment (4°C and 0°C). Data was extracted from publicly available RNASeq‐results (BioProject PRJNA602804) of three different hybrid sugar beet breeding lines (GT1, GT2, GT3) grown at 20°C and treated at 4°C and 0°C. Absolute expression values are presented as normalised counts per exon and million kilobases of reads (cpm) in a mean of *n* = 3 replicates per genotype and condition. The highest cpm values are marked in red, lowest in blue. White colour marks the 50 percentiles of the values in the shown data set. (C) Subcellular localisation of *Bv*INT1;1, *Bv*INT1;2, and *Bv*INT2‐fluorophor fusions. Representative confocal images of *A. thaliana* mesophyll protoplasts expressing *Bv*INT1;1 (left panel), *Bv*INT1;2 (middle panel), and *Bv*INT2 (right panel) as N‐terminal (upper line) or C‐terminal (middle and lower line) GFP fusions confocal single sections (upper and middle line) and maximal projections (lower line). Scale bars represent 10 µm.

In the Arabidopsis monosaccharide transporter (MST) gene family, four members show transport activity for inositol (*At*INT1 to *At*INT4; Schneider et al. 2008; Schneider et al. 2007; Schneider et al. 2006). Five sequences with homology to *A. thaliana* INTs were identified in the sugar beet genome (Dohm et al. 2014) (Figure 1A). Two of the corresponding gene products show the highest similarity to *At*INT1, the only tonoplast resident *At*INT, and were therefore named *Bv*INT1;1 and *Bv*INT1;2 (Figure 1A). To analyze gene transcript levels of those putative inositol transporters under cold‐ and freezing stress, we extracted expression values for the five INT genes from an RNASeq analysis performed on three different sugar beet genotypes (Figure 1B). The sugar beet genotypes GT1, GT2 and GT3, differed in their cold‐ and freezing tolerance, with GT1 being the most susceptible to cold‐ and freezing damage (Rodrigues et al. 2020; Keller, Müdsam, et al. 2021). RNASeq data suggested that one of the *At*INT1 homologues, *BvINT1;1*, was the highest expressed of all *At*INT homologues in all analyzed sugar beet genotypes even at 20°C (Figure 1B). Interestingly, when analyzing the expression of the different INT genes in leaf and taproot tissues of sugar beet plants grown at 20°C or in plants transferred to low temperatures (4°C and 0°C), *BvINT1;1* was the only isoform that showed a clear induction at low temperatures (Figure 1B). The expression of *BvINT1;1* in the taproot increased up to ninefold upon exposure to 4°C (Figure 1B), while the expression in leaves increased by a lesser extent, providing initial evidence that *BvINT1;1* plays an important role in the cold metabolism of sugar beet. It is interesting to mention that GT2, the sugar beet genotype with the highest cold‐ and freezing tolerance (Rodrigues et al. 2020; Keller, Müdsam, et al. 2021), exhibited the highest basal expression of *BvINT1;1* already at 20°C in both leaf‐ and taproot tissue (Figure 1B) and nearly double the expression level in the taproot when compared to the less tolerant genotypes GT1 and GT3 at 0°C (Figure 1B).

### 3.2 *Bv*INT1 Isoforms Localise to the Tonoplast

Further, we analyzed the subcellular localisation of different INT proteins in transiently transformed Arabidopsis protoplasts (Figure 1C). The GFP‐derived fluorescence of both N‐ and C‐terminal GFP fusions of *Bv*INT1;1 and *Bv*INT1;2 proteins formed a distinct thin line around transformed protoplasts with invaginations around the chloroplasts, which has been typically observed in vacuolar membrane localisations (Wolfenstetter et al. 2012; Müdsam et al. 2018). In contrast, the fluorescence of GFP‐INT2 and INT2‐GFP fusions could be assigned to the plasma membrane (Figure 1C). Co‐localisation studies with the tonoplast marker *At*NRAMP4‐RFP (Müdsam et al. 2018) showed an overlap of green *Bv*INT1;1‐GFP‐derived and red NRAMP4‐RFP‐derived fluorescence in vacuoles of *Bv*INT1;1‐GFP/NRAMP4‐RFP co‐transformed, but not in *Bv*INT2‐GFP/NRAMP4‐RFP protoplasts (Supporting Information S1: Figure S2A‐C). Due to the proximity of the tonoplast and the plasma membrane in isolated protoplasts, the red RFP‐derived and green GFP‐derived fluorescence for INT1;2 and INT2 respectively appear to partially merge, but analysis of the fluorescence signals near the chloroplast resolves the distinct fluorescence at the membranes surrounding the different compartments, that is, the vacuole and the cytosol (as indicated by the red and green arrows in Supporting Information S1: Figure S2C). However, to unequivocally confirm a tonoplast location of *Bv*INT1;1 a mild osmotic lysis of *Bv*INT1;1‐GFP/NRAMP4‐RFP co‐transformed Arabidopsis protoplasts was performed resulting in the release of vacuoles (Supporting Information S1: Figure S2D). There an overlap of the *Bv*INT1;1‐GFP derived fluorescence and the NRAMP‐RFP signal surrounding the released vacuole could be observed (Supporting Information S1: Figure S2D).

### 3.3 A Conserved C‐Terminal Dileucine‐Based Motif Is Necessary for Sorting of *Bv*INT1;1 to the Tonoplast

Sorting of vacuolar carrier proteins, for example, the copper transporter COPT5 (Klaumann et al. 2011) or the Arabidopsis inositol transporter INT1 (Schneider et al. 2008) requires a non‐classical di‐leucine‐based motif in the cytosolic C‐terminus of the protein. Accordingly, a leucine to alanine exchange results in miss‐sorting of mutated proteins to the plasma membrane (Wolfenstetter et al. 2012; Gasber et al. 2011).

We discovered a putative di‐leucine‐based motif at amino acid position 489 within the C‐terminal region of the *Bv*INT1;1 protein (Supporting Information S1: Figure S3A). In addition to a leucine pair (or leucine‐isoleucine), a characteristic feature of functional di‐leucine motifs is an acidic amino acid (glutamate or aspartate) upstream of ( = at position −4 from) the first leucine ([DE]XXXL[LI]) (Bonifacino and Traub 2003; Pedrazzini et al. 2013). A less strict [DE]X3‐5L[LI] consensus was proposed (Komarova et al. 2012). The corresponding ‘ENSQSLL’ sequence of *Bv*INT1;1 fulfilled the requirements of the latter consensus motif. To test the functionality of the putative di‐leucine‐based motif of *Bv*INT1;1, we exchanged the leucine‐ for an alanine‐pair and examined the subcellular localisation of the corresponding GFP‐fusion protein in Arabidopsis mesophyll protoplasts. As shown in Supporting Information S1: Figure S3B, *Bv*INT1;1^LL489/490AA^ ‐GFP indeed labels the plasma membrane of mesophyll protoplasts (*Arabidopsis thaliana)*, demonstrating that the C‐terminal di‐leucine was required for targeting *Bv*INT1;1 to the vacuolar membrane.

### 3.4 The BvINT1;1 Protein Shows High Specificity for the Transport of Inositol

**Figure 2:**
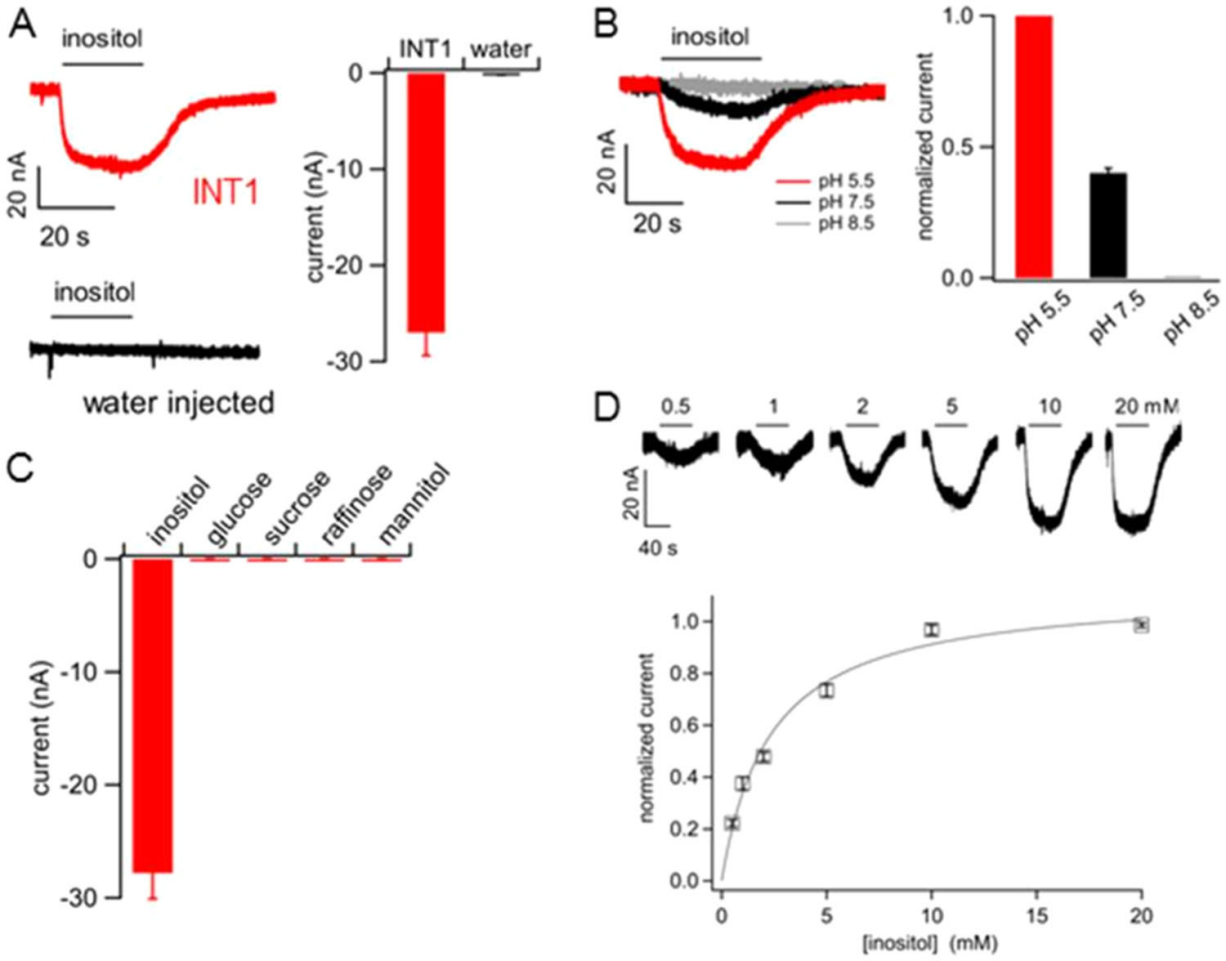
Functional analysis of *Bv*INT1;1 in *Xenopus laevis* oocytes. (A) Left: Membrane currents recorded in a *BvINT1;1* ^*PM*^ cRNA‐injected oocyte (red trace) and in a water‐injected oocyte (black trace), in response to exposure to 10 mM *myo*‐inositol (horizontal bar) in standard bath solution (pH 5.5), at a holding potential of −20 mV. Right: Summary plot of current amplitudes recorded in *BvINT1;1* ^*PM*^ cRNA‐injected oocytes (*n* = 18) and water‐injected oocytes (*n* = 7). (B) Left: Membrane currents recorded in a *BvINT1;1* ^*PM*^ cRNA‐injected oocyte, in response to exposure to 10 mM *myo*‐inositol (horizontal bar) in bath solutions adjusted to pH 5.5 (red trace), pH 7.5 (black trace) or pH 8.5 (grey trace). For representation purposes, traces were superimposed at the same baseline level. Holding potential was −20 mV. Right: Summary plot of current amplitudes recorded at pH 7.5 (*n* = 5 oocytes) and pH 8.5 (*n* = 3), normalised to the amplitudes recorded at pH 5.5. (C) Summary plot of current amplitudes in response to exposure to *myo*‐inositol (*n* = 18 oocytes), d‐glucose (*n* = 5), sucrose (*n* = 6), d‐raffinose (*n* = 6) or d‐mannitol (*n* = 3), each at 10 mM. (D) Top: Membrane currents recorded in a *BvINT1;1* ^*PM*^ cRNA‐injected oocyte, in response to exposure to increasing concentrations of *myo*‐inositol, as indicated, in standard bath solution (pH 5.5), at a holding potential of −20 mV. Bottom: dose–response analysis of normalised inositol‐induced currents. For each oocyte, current amplitudes were plotted against the applied inositol concentration, subjected to a Michaelis–Menten fit (see Material and Methods), and normalised to the maximum value at saturating inositol concentrations. The average half‐saturating concentration was 2.3 ± 0.3 mM (*n* = 7 oocytes). Data represent mean ± SE throughout the figure.

To gain further insights into its physiological role, we expressed *BvINT1;1* heterologous in *Xenopus laevis* oocytes and analyzed its transport properties using the two‐electrode voltage‐clamp technique (Schneider et al. 2008; Picco et al. 2015; Nieberl et al. 2017). To promote trafficking to the oocyte plasma membrane in these analyses, we used the INT1;1 variant in which both leucine residues of the C‐terminal dileucine motif had been exchanged for alanine (*Bv*INT1;1^LL489/490AA^, short INT1;1^PM^) (Supporting Information S1: Figure S3B). Exposure of *INT1;1* ^*PM*^ cRNA‐injected oocytes to 10 mM *myo*‐inositol elicited small inward currents, which were absent in water‐injected control oocytes (Figure 2A). The amplitudes of these inositol‐evoked currents were highest at pH 5.5 in the bath solution and strongly decreased at higher pH values. At pH 8.5, no transport activity could be detected (Figure 2B). This marked pH dependence suggested that inositol‐elicited inward currents were mediated by co‐transported protons, in line with an inositol/H^+^‐symport mechanism postulated for other INT family members (Schneider et al. 2006; Schneider et al. 2007). To test substrate specificity, INT1;1^PM^‐expressing oocytes were successively incubated in different sugars and sugar alcohols, and the resulting currents were recorded. In contrast to *myo*‐inositol, no current deflections were observed upon bath application of d‐glucose, sucrose, d‐raffinose, or d‐mannitol (Figure 2C), or a series of other tested substrates (d‐sorbitol, l‐rhamnose, d‐fucose, d‐arabitol, d‐mannose, d‐xylose, d‐ribose, d‐cellobiose, d‐melibiose; each at 10 mM; data not shown), indicating a high specificity of INT1;1^PM^ for the import of inositol into the cytosol. dose–response analyses showed that inositol‐dependent inward currents were already detectable at 0.5 mM and saturated at concentrations above 10 mM (Figure 2D). Fitting of the normalised current data with a Michaelis–Menten function yielded a half‐maximal concentration of 2.3 ± 0.3 mM (Figure 2D), characterising INT1;1 as a medium‐to‐low affinity transporter for inositol.

### 3.5 Inositol Contents and Metabolism Are Impaired in *Bvint1;1* Under Cold Treatment

To further investigate the function of *Bv*INT1;1, sugar beet mutants with an early stop codon at amino acid position 188 of *Bv*INT1;1 have been identified (Supporting Information S1: Figure S4A). The early stop codon is expected to lead to a truncated *Bv*INT1;1 protein in the process of translation. In comparison to functional *Bv*INT1;1, showing 12 α‐helical structures forming transmembrane domains (TMDs), the truncated version is predicted to only form five of those structures (Supporting Information S1: Figure S4B) rendering it non‐functional for pore formation and therefore transport of substrates across membranes. The corresponding lines with an early stop in *Bv*INT1;1 were backcrossed and then self‐crossed to generate homozygous, heterozygous, and wild type allele‐carrying segregants. For further analyses, homozygous (*bvint1;1*) and wild type allele‐carrying segregants (WT) were used, assuming that both lines, as direct sister lines, have the same genetic background except for the mutation in *Bv*INT1;1, thus providing reliable information on the influence of the absence of functional *Bv*INT1;1 in the plant's metabolism.

**Figure 3:**
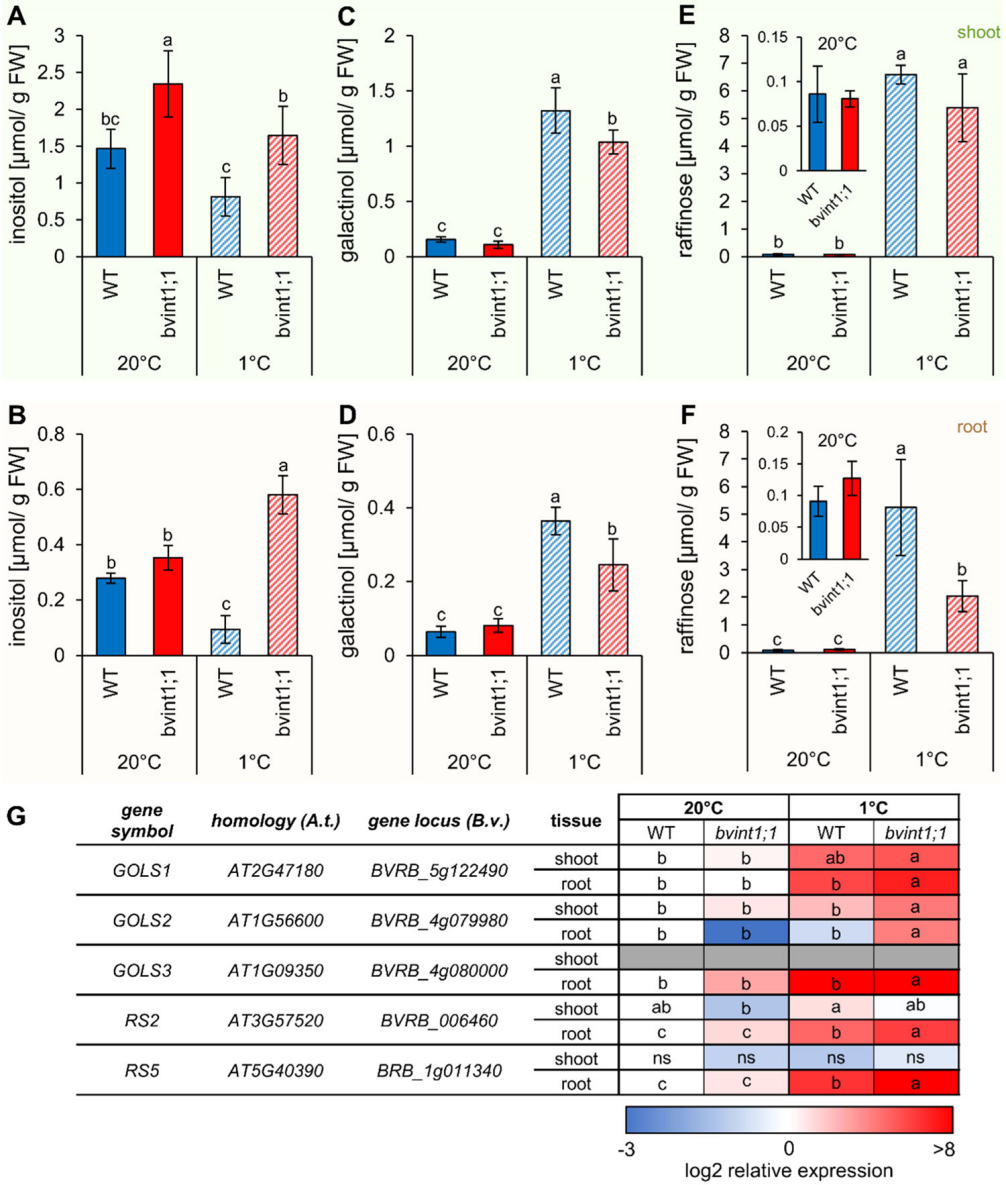
Contents and expression of enzymes involved in inositol and raffinose family oligosaccharides synthesis in wild type and *bvint1;1* sugar beet shoot and root. (A and B) Inositol, (C and D) galactinol and (E and F) raffinose contents in µmol/g FW in shoot (A, C, E) and taproot tissue (B, D, F) of wild type and *bvint1;1* sugar beet plants grown under standard conditions and at 1°C. Datapoints represent the mean of four biological replicates ±SDEV. (G) Heat map representation of the expression of the major sugar beet galactinol synthase (GOLS) and raffinose synthase (RS) genes in shoot and taproot tissue of wild type and *bvint1;1* sugar beet plants grown under standard conditions and treated at 1°C. The expression is given as log2 expression relative to the expression in the wild type at 20°C. Colour coding indicates lower expression (blue) or higher expression (red) of the corresponding gene in relation to the wild type at 20°C. Grey bars represent non‐detectable expression values. Different letters indicate significant differences between the different lines and conditions according to two‐way ANOVA with post hoc Tukey testing (*p* < 0.05).

First, we analyzed the effects of lacking *Bv*INT1;1 on inositol contents of shoot and root tissues (Figure 3A,B). Already under standard conditions, inositol contents were significantly higher in the shoots of *bvint1;1* plants in comparison to the wild type (Figure 3A). In a large batch of plants from a segregating population representing *bvint1;1*/*bvint1;1* (homoz. *bvint1;1*), *Bv*INT1;1/*bvint1;1* (heteroz. *bvint1;1*) and *Bv*INT1;1/*Bv*INT1;1 (wild type, WT) plants, the inositol contents in the shoots of all identified *bvint1;1*/*bvint1;1* plants were higher than in *Bv*INT1;1/*Bv*INT1;1 and most of the heterozygous *Bv*INT1;1/*bvint1;1* plants (Supporting Information S1: Figure S5). This strongly suggested that the high inositol contents were associated with the mutated *bvint1;1* locus (Supporting Information S1: Figure S5). After exposure to low temperatures (1°C) for 3 weeks, inositol contents in the shoots of wild type and *bvint1;1* were lower than under control conditions. However, *bvint1;1* plants still showed significantly higher inositol contents than the wild type (Figure 3A).

Inositol contents were approximately 50% lower in taproot tissue than in shoots under standard conditions (Figure 3A,B). Consequently, inositol contents were slightly higher in *bvint1;1* than in the wild type (Figure 3B). Similar to the shoot, cold exposure resulted in a decline of inositol levels in taproots of the wild type (Figure 3B). In sharp contrast to that, cold exposure led to a strong increase of inositol levels in taproots of *bvint1;1*, resulting in significantly higher concentrations in comparison to the wild type under cold conditions (Figure 3B). These results together with the proposed activity of *Bv*INT1;1 as a vacuolar inositol exporter indicated that inositol was trapped in vacuoles of *bvint1;1* plants, especially after cold treatment, when *Bv*INT1;1 gene expression was induced in taproots (Figure 1B). Results from the analysis of inositol levels in isolated taproot vacuoles of wild type and *bvint1;1* plants supported such a scenario. Here, inositol levels of the isolated vacuole fraction of *bvint1;1* taproots were higher than that of wild type vacuoles already at control conditions (Supporting Information S1: Figure S6).

Inositol is an important substrate for the biosynthesis of galactinol and further RFOs like raffinose, which serve as important osmolytes and ROS‐quenchers under abiotic stress conditions like cold (Keller, Müdsam, et al. 2021). Therefore, contents of both galactinol and raffinose were determined in shoot and taproot samples of wild type and *bvint1;1* under control and cold conditions (Figure 3D–G). Concentrations of galactinol and raffinose were comparable between wild type and *bvint1;1* under standard conditions independent of the analyzed tissue (Figure 3C–F). Cold treatment led to a significant increase of galactinol and most markedly of raffinose in both shoot and root tissues of *bvint1;1* and wild type plants. However, cold‐dependent accumulation of the two compounds in taproots of *bvint1;1* was less pronounced than in the wild type. Especially raffinose levels only reached about 35% of the wild type level in *bvint1;1* taproots in the cold, suggesting that inositol is trapped in the taproot vacuoles of *bvint1;1*. Trapping of vacuolar inositol consequently suggests a reduced cytosolic availability of the metabolite in *bvint1;1* plants and therefore results in a reduced cold‐dependent raffinose biosynthesis in the cytosol of the taproot (Figure 3C–F).

To check whether the lower galactinol and raffinose levels in *bvint1;1* could be due to lower expression of genes coding for galactinol and raffinose synthesising enzymes, we analyzed the expression of the three major galactinol synthase (*GOLS1‐3*) and of the two Raffinose synthase genes (*RS2* and *RS5*) (Keller, Müdsam, et al. 2021) in wild type and *bvint1;1* under control and cold conditions (Figure 3G, Supporting Information S2: Table S2). GOLS and RS are cytosolic enzymes catalysing the synthesis of galactinol from UDP‐galactose and inositol (GOLS), or of raffinose from galactinol and sucrose (RS). The expression of the respective genes increased after cold treatment, especially in taproots. Expression of *GOLS1‐3*, *RS2*, and *RS5* in *bvint1;1* taproots was higher than in wild type taproots suggesting a compensatory mechanism because of reduced inositol availability for galactinol and raffinose biosynthesis in the cytosol (Figure 3G, Supporting Information S2: Table S2).

### 3.6 *Bvint1;1* Sugar Beets Show Impaired Biomass Accumulation and Photosynthetic Activity Under Cold Treatment

**Figure 4:**
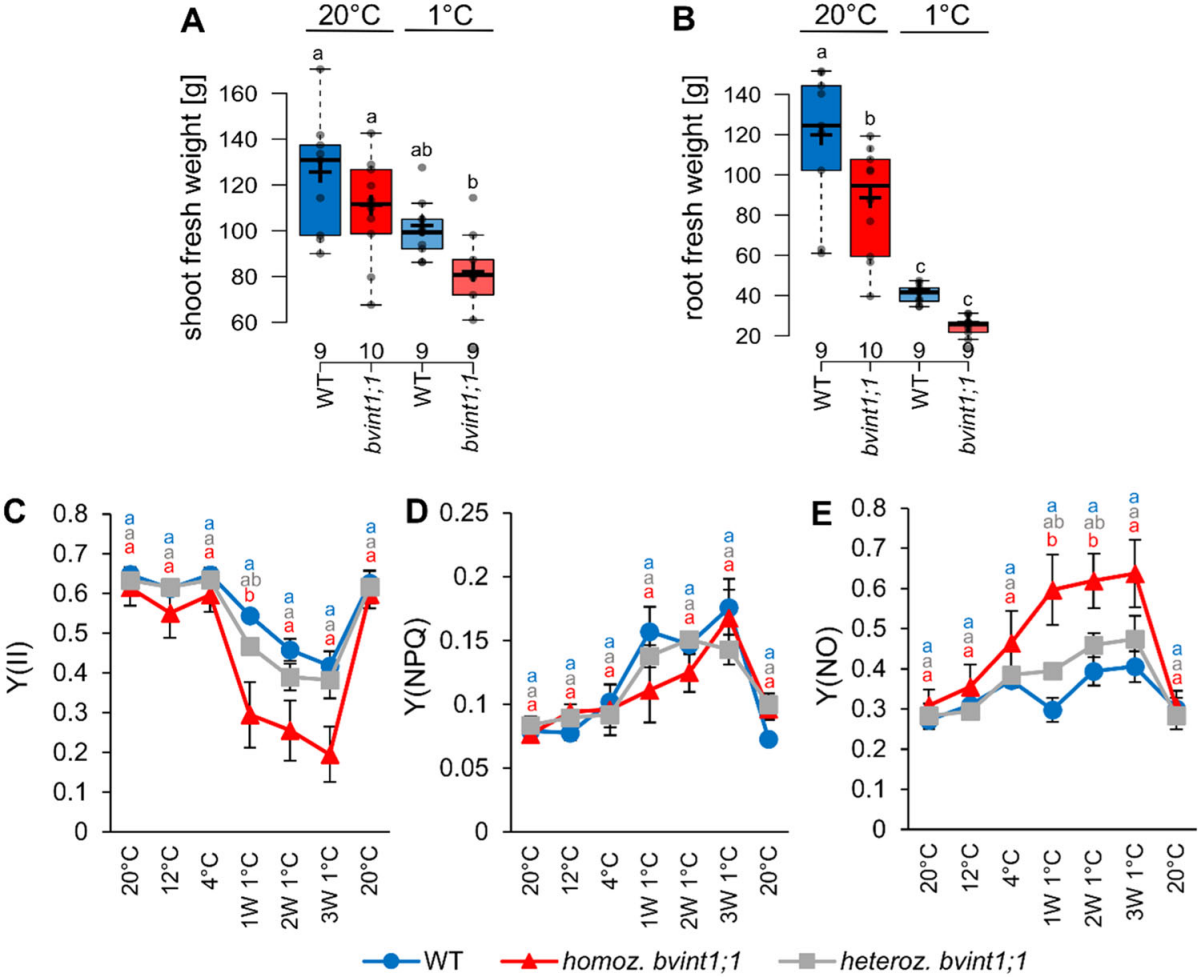
Biomass accumulation and photosynthetic parameters of wild type and *bvint1;1* sugar beet plants under ambient and cold growth temperatures. (A) Shoot and (B) Root fresh weight of wild type and *bvint1;1* grown at 20°C and plants treated at 1°C for three weeks determined over nine to ten individual plants. (C) Quantum yield of photosynthesis [Y(II)], (D) regulated nonphotochemical energy loss [Y(NPQ)], and (E) unregulated nonphotochemical energy loss [Y(NO)] of wild type and heterozygous and homozygous *bvint1;1* plants grown at 20°C for 15 weeks and stepwise exposure to 12°C, 4°C, 1°C and recovery at 20°C after cold treatment. Datapoints represent the mean over three biological replicates ±SE. Different letters indicate significant differences between the different lines and conditions according to one‐way ANOVA with post hoc Tukey testing (*p* < 0.05) in (A and B) and two‐way ANOVA with post hoc Tukey testing (*p* < 0.05) in (C–E).

Lack of functional *Bv*INT1;1 resulted in increased inositol contents and reduced raffinose levels especially in sugar beet taproots under cold treatment (Figure 3). To analyze whether this also affects biomass accumulation, sugar beet plants were weighed, and photosynthetic parameters were determined. The absence of functional *Bv*INT1;1 in *bvint1;1* plants resulted in slightly reduced leaf and significantly reduced root biomass compared to the wild type when grown under standard conditions (Figure 4A,B). Three weeks of cold treatment provoked a reduced shoot and root biomass in comparison to plants kept at control conditions (Figure 4A,B). While root biomass was significantly reduced in both wild type and *bvint1;1* after cold treatment, with *bvint1;1* roots showing the lowest root biomass, the shoot biomass only was significantly reduced due to cold treatment in *bvint1;1* plants (Figures 4A,B). The reduced biomass of *bvint1;1* plants especially under cold treatment was accompanied by a significant reduction in the yield of Photosystem II (YII) (Figure 4C). While Y(II) was unaltered in wild type, heterozygous and homozygous *bvint1;1* knockout plants at 20°C, 12°C and 4°C, the parameter decreased at 1°C treatment, with a significant reduction in homozygous *bvint1;1* plants (Figure 4C). However, when plants were transferred to 20°C after cold treatment, Y(II) was restored to initial levels (Figure 4C). Compared to Y(II), Y(NPQ) tends to increase during cold treatment in all lines, but not significantly, while Y(NO) increased significantly in homozygous *bvint1;1* plants during 1°C treatment (Figures 4D,E). Thus, Y(NO) in *bvint1;1* plants was not only significantly increased at 1°C compared to 20°C treatment, but also compared to the wild type under similar conditions (Figure 4E).

### 3.7 Sugar Contents Differ Between Wild Type and *Bvint1;1* Shoot and Taproot Tissues Under Cold Treatment

Sugar beet stores high levels of the disaccharide sucrose in taproot tissues and the distribution and accumulation of this sugar is highly affected by cold treatment (Rodrigues et al. 2020). Because the above results showed that lack of *Bv*INT1;1 influenced cold‐dependent metabolic processes and biomass accumulation, we were interested in analyzing the content of sucrose as the main carbon storage compound in taproots together with that of the sucrose building blocks glucose and fructose and starch. Under control conditions, both the shoot as well as the taproot of *bvint1;1* showed slightly but not significantly elevated levels of glucose and fructose but not of sucrose compared to the wild type (Supporting Information S1: Figure S7A–F). Starch levels were significantly lower in *bvint1;1* shoots in comparison to the wild type at 20°C (Supporting Information S1: Figure S7G).

In the taproot tissue, the opposite behaviour could be observed, as *bvint1;1* plants showed slightly but not significantly higher starch contents than present in wild type (Supporting Information S1: Figure S6H). When exposed to cold treatment for 3 weeks, glucose, fructose, and sucrose contents accumulated in leaves of the wild type but not of *bvint1;1*, leading to significantly higher levels of those metabolites in the wild type under cold (Supporting Information S1: Figure S7A,C,E). In contrast to that, starch levels significantly decreased to comparable levels in both lines under cold treatment (Supporting Information S1: Figure S6G). In the root tissue, significantly higher amounts of glucose could be measured in *bvint1;1* plants in the cold in comparison to the wild type, resulting from the significant accumulation of glucose upon the cold stimulus in *bvint1;1* (Supporting Information S1: Figure S7B). Fructose contents however, were comparable in the root tissue of all lines under the different temperature conditions (Supporting Information S1: Figure S7D). Sucrose contents in the root did not differ between wild type and *bvint1;1* under neither control nor cold conditions, however, wild types showed a significant decrease of root sucrose under cold treatment (Supporting Information S1: Figure S7F). For starch levels, as observed in the shoot, a significant decrease to comparable levels was observed in both analyzed lines upon cold treatment in the taproot (Supporting Information S1: Figure S7H). Overall, *bvint1;1* plants accumulated fewer sugars (glucose, fructose, and sucrose) in the leaves than wild type plants under cold treatment, while contents of such sugars were slightly higher in *bvint1;1* taproots than in the wild type during cold (Supporting Information S1: Figure S7).

### 3.8 *Bvint1;1* Shows High Need For ROS Detoxification in the Taproot

**Figure 5:**
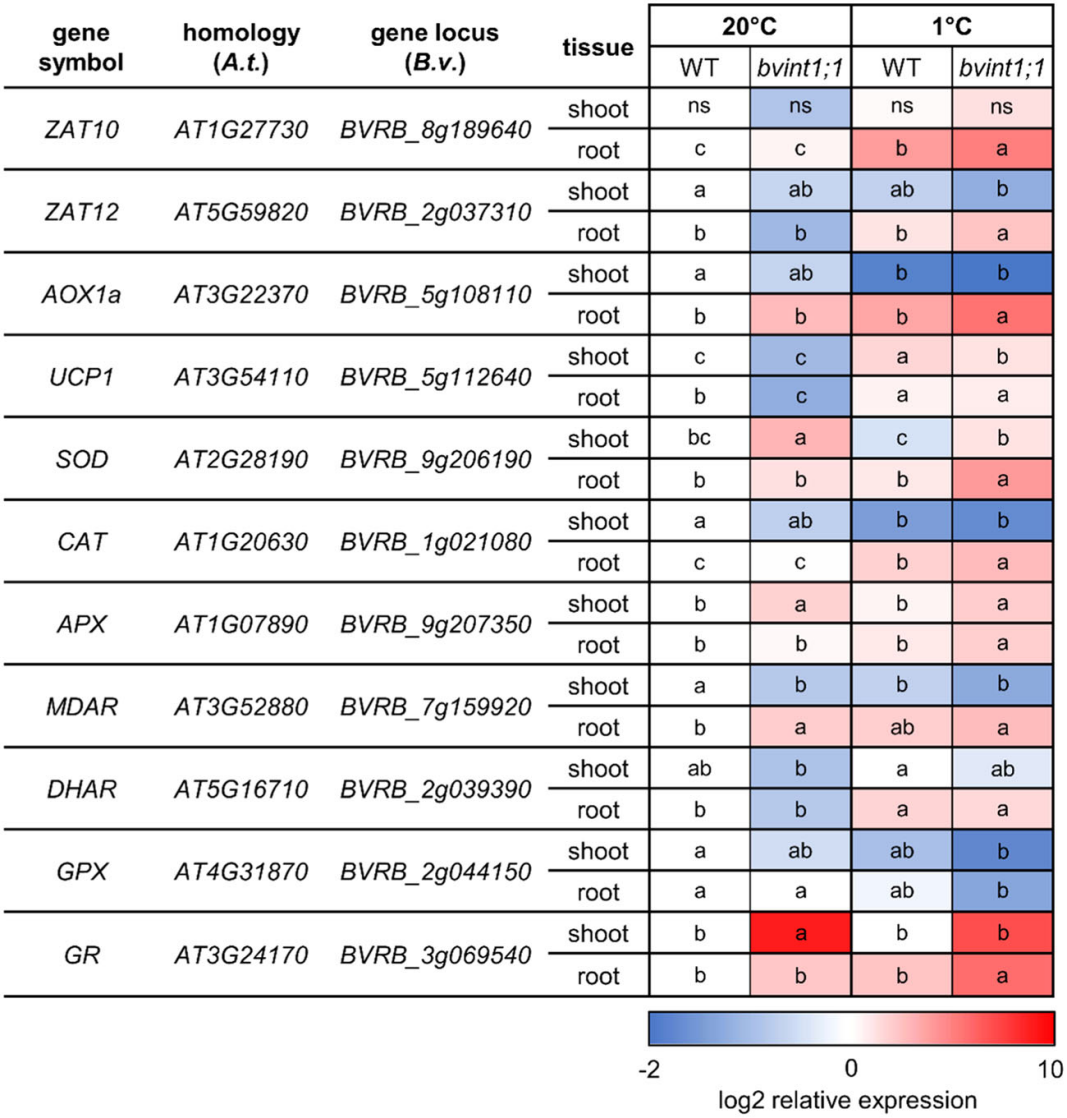
Expression of ROS‐Marker Enzymes in wild type and *bvint1;1* sugar beet shoot and root. Heat map representation of the expression of ROS marker enzymes in shoot and taproot tissue of wild type and *bvint1;1* sugar beet plants grown under standard conditions and treated at 1°C. The expression is given as log2 expression relative to the expression in the wild type at 20°C. Colour coding indicates lower expression (blue) or higher expression (red) of the corresponding gene in relation to the wild type at 20°C. Different letters indicate significant differences between the different lines and conditions according to two‐way ANOVA with post hoc Tukey testing (*p* < 0.05).

*Bvint1;1* plants show reduced raffinose contents (Figure 3E,F), especially in the taproot tissue, lower biomass, and increased Y(NO) (Figure 4A,B,E) under cold treatment. All these findings can be linked to increased accumulation of ROS in those plants. To check this, we analyzed the expression of genes involved in cellular ROS metabolism and detoxification (Keller, Müdsam, et al. 2021) (Figure 5, Supporting Information S2: Table S3). These comprised the two ROS‐responsive transcription factors ZAT10 and ZAT12 (Mittler et al. 2006; Davletova et al. 2005), the Alternative Oxidase 1a (AOX1a) and the Uncoupling Protein 1 (UCP1), which is involved in the prevention of ROS production in mitochondria (Borecký and Vercesi 2005; Fuchs et al. 2022). Further, the expression of genes encoding the ROS detoxifying enzymes Superoxide Dismutase (SOD) and Catalase (CAT), and the enzymes of the Foyer‐Halliwell ascorbate‐glutathione cycle like Ascorbate Peroxidase (APX), Monodehydroascorbate Reductase (MDAR), Dehydroascorbate Reductase (DHAR), Glutathione Peroxidase (GPX) and Glutathione Reductase (GR) were analyzed (Baek und Skinner, 2012; Mittler et al. 2004; Foyer and Noctor 2011). In general, the transcription of several ROS‐related genes was significantly induced in control conditions in the mutant compared to the wild type (e.g., SOD, APX and GR in shoot and MDAR in root; Figure 5). Exposure to cold temperatures for 3 weeks led to a significant upregulation of some of the ROS markers in the shoot (e.g., *UCP1*) and a majority of the ROS markers (e.g., *ZAT10, UCP1, CAT, DHAR*) in the taproot of both wild type and *bvint1;1* (Figure 5). Nevertheless, in a direct comparison between wild type and *bvint1;1*, several genes were significantly higher in shoots, for example, *SOD*, *APX*, and in roots e.g., *ZAT10*, *ZAT12*, *AOX1a*, *SOD*, *CAT*, *APX*, *GR* of *bvint1;1* plants under cold conditions (Figure 5, Supporting Information S2: Table S3). Overall, expression analysis revealed the highest expression of ROS marker genes in the taproot of *bvint1;1* under cold conditions (Figure 5). This data, in summary, suggested that *bvint1;1* plants experienced higher ROS‐related responses in comparison to the wild type.

## 4 Discussion

Given that (i) sugar‐beet taproot metabolism and gene expression are highly responsive to low temperatures (Klotz and Haagenson 2008; Rodrigues et al. 2020; Keller, Müdsam, et al. 2021), (ii) inositol metabolism and raffinose biosynthesis are induced by low temperatures in sugar beet (Keller, Müdsam, et al. 2021), and (iii) inositol is compartmentalised between the cytosol and the vacuole (Schneider et al. 2008), temperature‐regulated vacuolar inositol transport processes must be active for efficient biosynthesis of protective inositol‐dependent compounds galactinol and raffinose.

In contrast to Arabidopsis, where four different tonoplast and plasma membrane‐localised inositol transporters have been described (Schneider et al. 2006, 2007, 2008), the genome of *Beta vulgaris* encodes five proteins with high homology to Arabidopsis inositol transporters (Figure 1A). Of these, two proteins designated *Bv*INT1;1 and *Bv*INT1;2, which share 78% sequence identity, show high homology to the Arabidopsis vacuolar inositol exporter *At*INT1 (Figure 1A). Similar observations could also be made for the two homologues of the vacuolar sucrose transporter from Arabidopsis *At*TST2 in *Beta vulgaris*. In that case, *Bv*TST2;1 and *Bv*TST2;2 show high sequence identity but only *Bv*TST2;1 is the major vacuolar sucrose loader mediating high sucrose accumulation in the taproot (Jung et al. 2015). Among all five *At*INT homologues, transcripts for *BvINT1;1* and *BvINT1;2* exhibited the highest expression in taproots and shoots, but only the *BvINT1;1* gene shows a strong transcriptional induction upon cold treatment, especially in the taproot tissue (Figure 1B). Therefore, the high demand for inositol for cold acclimation processes, such as galactinol and further raffinose synthesis (Taji et al. 2002; Saito and Yoshida 2011), and the marked cold‐induced upregulation of *BvINT1;1* expression, especially in the sugar beet taproot where ROS generation via respiratory processes is pronounced (Keller, Müdsam, et al. 2021, Rodrigues et al. 2020), underline the importance of inositol transport processes in the cold metabolism of sugar beet. Interestingly, expression of *BvINT1;1* upon cold treatment (0°C) was highest in taproots of a sugar beet genotype (GT2; Figure 1B), which shows high cold‐ and frost tolerance and specifically high raffinose concentrations in the taproot pith (Keller, Müdsam, et al. 2021) further supporting the importance of inositol transport processes in the *Beta vulgaris* cold response. Further, in a recent analysis of genomic variation in the Beta genus, *BvINT1;1* was found to be predominantly homozygous for cultivated and rather cold‐sensitive reference haplotypes, whereas high variation was observed among generally more cold‐tolerant wild beet ancestors (Felkel, Dohm, and Himmelbauer 2023). However, a direct connection between the high variation in *BvINT1;1* and better abiotic stress tolerance in wild beet ancestors remains to be proven.

Based on subcellular localisation analysis using GFP‐constructs (Figure 1C, Supporting Information S1: Figure S2) and two‐electrode voltage‐clamp electrophysiology performed on *BvINT1;1* expressing Xenopus oocytes (Figure 2), *Bv*INT1;1 was characterised as a highly specific tonoplast‐located inositol transporter, exporting inositol in symport with a proton out of the vacuole. Such transport characterisation of *Bv*INT1;1 was possible due to targeting of the protein to the plasma membrane instead of the tonoplast after mutation of a C‐terminal dileucine motif (Supporting Information S1: Figure S2). Thereby, in either the tonoplast or plasma membrane isoform C‐ and N‐terminus of the protein are expected to face the cytosol. Given the proton gradient over the membranes, inositol is expected to be transported in a symport with a proton from the vacuole to the cytosol in the tonoplast‐located isoform. On the other hand, the plasma membrane located BvINT1;1^PM^ is mediating an import of inositol from the apoplast to the cytosol of the cell, driven by an inward‐facing pH gradient. The function of *Bv*INT1;1 as an inositol‐proton symporter might further suggest regulation of its transport activity by posttranslational modifications, as otherwise, the pH gradient across the tonoplast would drive its transport substrate almost completely out of the vacuole. Indeed, posttranslational modifications regulating transport activities are common in plant MST transporters, for example, various phosphorylation sites could be found in Tonoplast Sugar Transporter (TST) homologues (Whiteman et al. 2008; Endler et al. 2009) and first kinases mediating mainly stress‐induced phosphorylation of these sites were identified (Wingenter et al. 2011; Deng et al. 2020). As *Bv*INT1;1, similar to TST proteins, also harbours a big cytosolic loop connecting TMD six and seven, possible phosphorylation in this region seems likely.

However, the origin of such high vacuolar inositol concentrations meeting the described transport characteristics of *Bv*INT1;1 in adult sugar beet plants is not entirely clear. To date, no vacuolar inositol importer has been described in any plant species. Anyhow, it is very likely that inositol is transported to the vacuole in the form of the sixfold phosphorylated inositol hexakisphosphate, also known as phytate. Phytate represents an important storage compound for phosphate in plant seedlings (Silva et al. 2021) and is transported into the vacuole via the ABC‐transporter MRP5 (Nagy et al. 2009) or, alternatively, reaches the vacuole via vesicle transport from the Endoplasmic Reticulum (Greenwood and Bewley 1984; Otegui, Capp, and Staehelin 2002). Further, inositol can enter the vacuole in the form of phosphatidylinositol 3‐phosphate, a phospholipid with a regulatory role in vesicle trafficking (Kim et al. 2001). Therefore, vacuolar inositol likely derives from the breakdown of phytate or membrane compounds and is a product of inositol recycling processes (Schneider et al. 2008; Schneider 2015).

The export of recycled vacuolar inositol to the cytosol seems to be of importance for proper plant development, as it can be seen in (i) severe retardation of root growth in *atint1;1* mutant plants (Schneider et al. 2008) and (ii) the reduced taproot biomass in *bvint1;1* sugar beet mutant plants (Figure 4B). Such reduced growth in *atint1;1* mutants was attributed to reduced cell elongation in a sucrose‐dependent manner (Strobl et al. 2018). However, the link between sucrose and inositol could not be determined, yet. The only metabolic connection between inositol and sucrose can be seen in the synthesis of raffinose. This is because cytosolic inositol serves as a substrate for galactinol synthesis, which subsequently leads to the synthesis of raffinose in a reaction with sucrose. However, since there is only one isoform of the vacuolar inositol exporter in Arabidopsis, but two in sugar beet (*Bv*INT1;1 and *Bv*INT1;2), *Bv*INT1;2 could at least partially rescue the absence of *Bv*INT1;1, so comparisons between *atint1;1* and *bvint1;1* mutants must be treated with caution. This is illustrated by the fact that wild type and *bvint1;1* show no differences in raffinose or sucrose content under standard conditions, even when root growth is already impaired (Figure 3, Supporting Information S1: Figure S7).

Under cold treatment, such growth inhibition is even more pronounced. Under latter conditions *bvint1;1* mutants do not only show retarded taproot growth (Figure 4B) but especially impaired shoot development (Figure 4A). The retarded growth phenotype under cold treatment (Figures 4A,B), as well as the increased expression of *BvINT1;1* (Figure 1B) under cold stress underlines the importance of that transporter in the cold metabolism of the plant. Cold treatment, as well as other abiotic stress conditions, are known to induce the expression of the cytosolic RFO synthesising enzymes GOLS and RS. These enzymes metabolise UDP‐Galactose and inositol to galactinol and, in the next reaction with sucrose further lead to raffinose (Taji et al. 2002; Saito and Yoshida 2011; Li et al. 2020). This effect has also been observed on sugar beet plants (Figure 3G; Keller, Müdsam, et al. 2021). Correspondingly, trapping of inositol in vacuoles of sugar beets by a lack of functional *Bv*INT1;1 (Supporting Information S1: Figure S6) results in increased inositol contents and decreased contents of galactinol and raffinose, especially under cold treatment in the taproot (Figure 3A–F), even though the strong upregulation of *GOLS* and *RS* expression (Figure 3G). The high expression of these genes in *bvint1;1* underlines that the reduced galactinol and raffinose contents are solely due to a lack of substrate rather than a defect in transcriptional regulation. While *bvint1;1* mutants showed increased inositol contents during cold treatment (Figure 3A,B), the wild type exhibited a decreased inositol content under cold, underlining the importance and metabolic consumption of this compound in the plant's cold response. As RFOs like raffinose are important contributors in ROS‐scavenging processes, especially of the harmful hydroxyl radical (Nishizawa, Yabuta, and Shigeoka 2008; Matros et al. 2015; Pommerrenig et al. 2018), growth deficits, especially in the taproot of *bvint1;1* plants under cold treatment might be attributed to reduced ROS‐scavenging activity due to reduced RFO‐levels in these plants (Figures 3E,F).

Such increased ROS accumulation in *bvint1;1* mutants can not only be seen in the reduced growth capacity under cold treatment (Figure 4A,B) but also in the decreased Y(II) in leaves and increased Y(NO) under cold (Figure 4C–E), as well as in the increased expression of ROS‐detoxifying enzymes and the ROS‐marker genes *ZAT10* and *ZAT12* (Figure 5), which are induced in their expression upon increased ROS accumulation (Rizhsky et al. 2004; Scarpeci et al. 2008; Nguyen et al. 2016). Those findings concur with previous research showing that raffinose accumulation, especially in the pith region of the sugar beet taproot, positively correlates with the freezing tolerance of the plant, probably due to increased ROS‐scavenging capacities (Keller, Müdsam, et al. 2021). Further, not only in sugar beet plants but also in other plants like sweet potatoes and apples, a positive effect of cytosolic inositol availability on antioxidant activity was shown under different abiotic stress conditions (Zhai et al. 2016; Hu et al. 2020). Especially the findings in apple are interesting, as also in this plant a silencing of the vacuolar inositol transporter INT1 resulted in an increase in ROS under abiotic stress (Hu et al. 2020). However, it is worth mentioning that already under standard growth conditions higher expression of ROS markers (e.g., *SOD*, *APX* and *GR*) could be observed in the shoot tissues of *bvint1;1* (Figure 5) although no differences in the galactinol and raffinose contents compared to the wild type could be observed under these conditions (Figure 3C,E). This suggests an influence of inositol trapping not only on raffinose biosynthesis but also on metabolic shuttling of inositol to alternative pathways involved in ROS homoeostasis like the synthesis of the antioxidant ascorbate. Earlier, a connection between inositol availability and ascorbate synthesis was proposed as overexpression of a myo‐inositol oxygenase (MIOX) resulted in increased ascorbate levels in Arabidopsis (Lorence et al. 2004). However, the metabolic connection between inositol and ascorbate is controversially discussed (Endres and Tenhaken 2009).

Overall, our findings reveal an important role of the vacuolar inositol transporter *Bv*INT1;1 in sugar beet cold metabolism. The corresponding gene therefore represents a promising target for directed breeding strategies aimed at enhancing the cold tolerance of sugar beet plants. This is particularly important, given the shortened growth period of the plant in temperate climates, from late spring to early autumn, to circumvent periods of low temperatures which can cause a significant decrease in crop yield.

## Conflicts of Interest

O.C., D.H., W.K., and F.L. are employed by KWS SAAT SE & Co. KGaA. This study received funding from KWS SAAT SE & Co. KGaA. The funder had the following involvement with the study: selection and propagation of germplasm material and plant growth for cold treatment. The authors declare no conflicts of interest.

## Supporting information

Supporting information.

Supporting information.

## Data Availability

All data supporting the findings of this study are available within the article [and/or] its Supporting Information.
