# Supplementary material for: The Vacuolar Inositol Transporter BvINT1;1 Contributes to Raffinose Biosynthesis and Reactive Oxygen Species Scavenging During Cold Stress in Sugar Beet
Source: Plant Cell Environ. 2025 Jan 8;48(5):3471–86. doi: 10.1111/pce.15367 (PMC11963481; doi:10.1111/pce.15367)
Supplement: Supplementary file 1 — Supporting information. [file PCE-48-3471-s002.docx]

**Supplemental Figures S1-S6**

**The vacuolar inositol transporter *BvI*NT1;1 contributes to raffinose biosynthesis and reactive oxygen species scavenging during cold stress in sugar beet**

Johannes Berg^1^, Cristina Martins Rodrigues^1^, Claire Scheid^1^, Yana Pirrotte^1^, Cristiana Picco^2^, Joachim Scholz-Starke^2^, Wolfgang Zierer^3^, Olaf Czarnecki^4^, Dieter Hackenberg^4^, Frank Ludewig^4^, Wolfgang Koch^4^, H. Ekkehard Neuhaus^1^, Christina Müdsam^3^, Benjamin Pommerrenig^1§^, Isabel Keller^1*^

**Figure S1**: Schematic representation of the plant growth regime.

**Figure S2:** Subcellular localization analysis of *Bv*INT1;1 in Arabidopsis protoplasts.

**Figure S3:** A C-terminal dileucine motif is responsible for the tonoplast location of *Bv*INT1;1.

**Figure S4:** Characterization of *bvint1;1* mutants.

**Figure S5:** Inositol content in crude extract and isolated taproot vacuoles of wild types and *bvint1;1* under standard growth conditions.

**Figure S6:** Inositol content in crude extract and isolated taproot vacuoles of wild types and *bvint1;1* under standard growth conditions.

**Figure S7:** Glucose, fructose, sucrose, and starch contents in wild type and *bvint1;1* sugar beet shoot and root


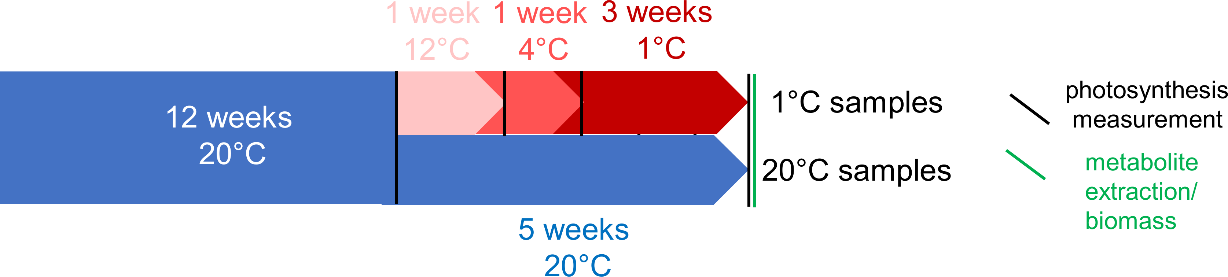


**Supplemental Figure 1: Schematic representation of the plant growth regime.**

Sugar beet plants were grown at 20°C for 12 weeks. Afterward, the population of plants was split into two halves. One half was transferred to 12°C and 4°C for one week, respectively and further temperature was lowered to 1°C for three weeks afterwards. The other half of the plants constantly stayed at 20°C as control. After total plant growth for 17 weeks, plants were harvested for biomass determination, metabolite extraction, and gene expression analysis, as indicated by the green line. For the determination of the photosynthetic activity, a kinetics analysis was performed. Chlorophyll autofluorescence was determined in weekly intervals during cold treatment, as indicated by the black line.


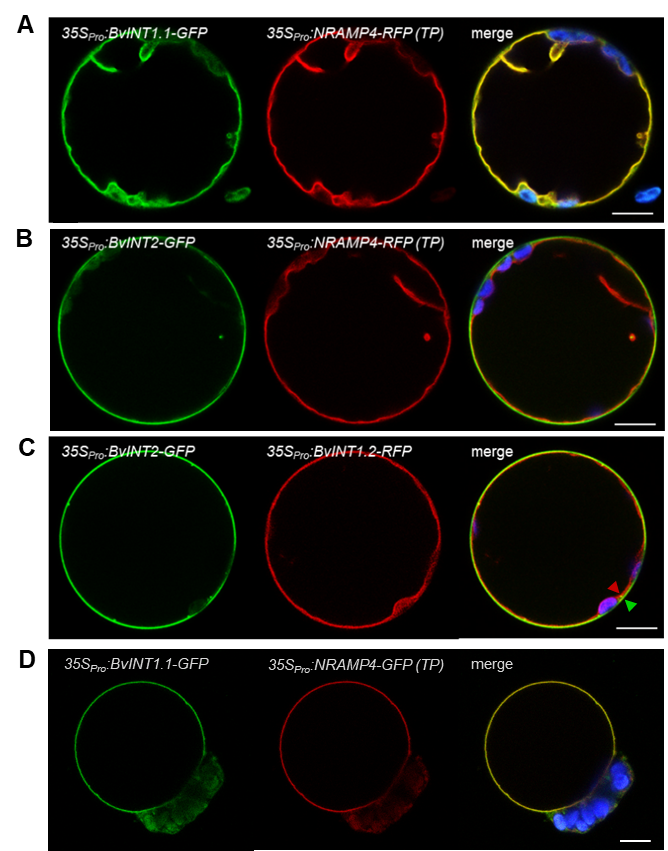


**Supplemental Figure 2: Subcellular localization analysis of BvINT1;1 in Arabidopsis protoplasts.**

Colocalization analysis of *Bv*INT1;1 (**A**) and *Bv*INT2 (**B**) C-terminal GFP fusions (left column) and the tonoplast NRAMP4-RFP (middle column). The merged signal of NRAMP and INT fluorescence fusion is shown in yellow and chlorophyll autofluorescence in blue (right column). **C**) Colocalization analysis of *Bv*INT2 C-terminal GFP fusion (left column) and *Bv*INT1;1 C-terminal RFP fusion (middle column). The merged GFP and RFP fluorescence signal is shown in yellow and chlorophyll autofluorescence in blue (right column). **D**) Mild osmotic lysis of Arabidopsis protoplasts expressing *Bv*INT1;1 C-terminal GFP fusion constructs (left column) and the tonoplast NRAMP4-RFP (middle column). The merged signal of NRAMP and INT fluorescence fusion is shown in yellow and chlorophyll autofluorescence in blue (right column). Scale bars represent 10µm.


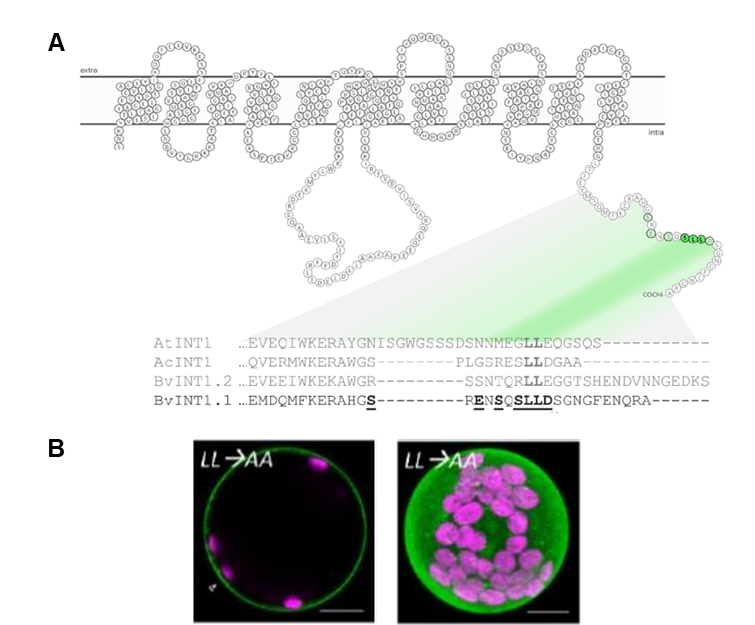


**Supplemental Figure 3: A C-terminal dileucine motif is responsible for the tonoplast location of *Bv*INT1;1.**

**A)** Consensus sequence and location of the C-terminal dileucine motif in the amino acid sequence of *Arabidopsis thaliana* INT1 (AtINT1), *Ananas comosus* INT1 (AcINT1) and the two *Beta vulgaris* INT1 isoforms (BvINT1;1 and BvINT1;2). The characteristic dileucine pair is marked in bold letters in all the corresponding INT-Sequences of the alignment. **B)** Subcellular localization of BvINT1;1 with a mutated dileucine motif (BvINT1;1^LL489/490AA^), where the characteristic dileucine was replaced by two alanines in a single section (left panel) and maximal projection (right panel). GFP-signal is shown in green, chlorophyll autofluorescence is in pink. Scale bars represent 10µm.


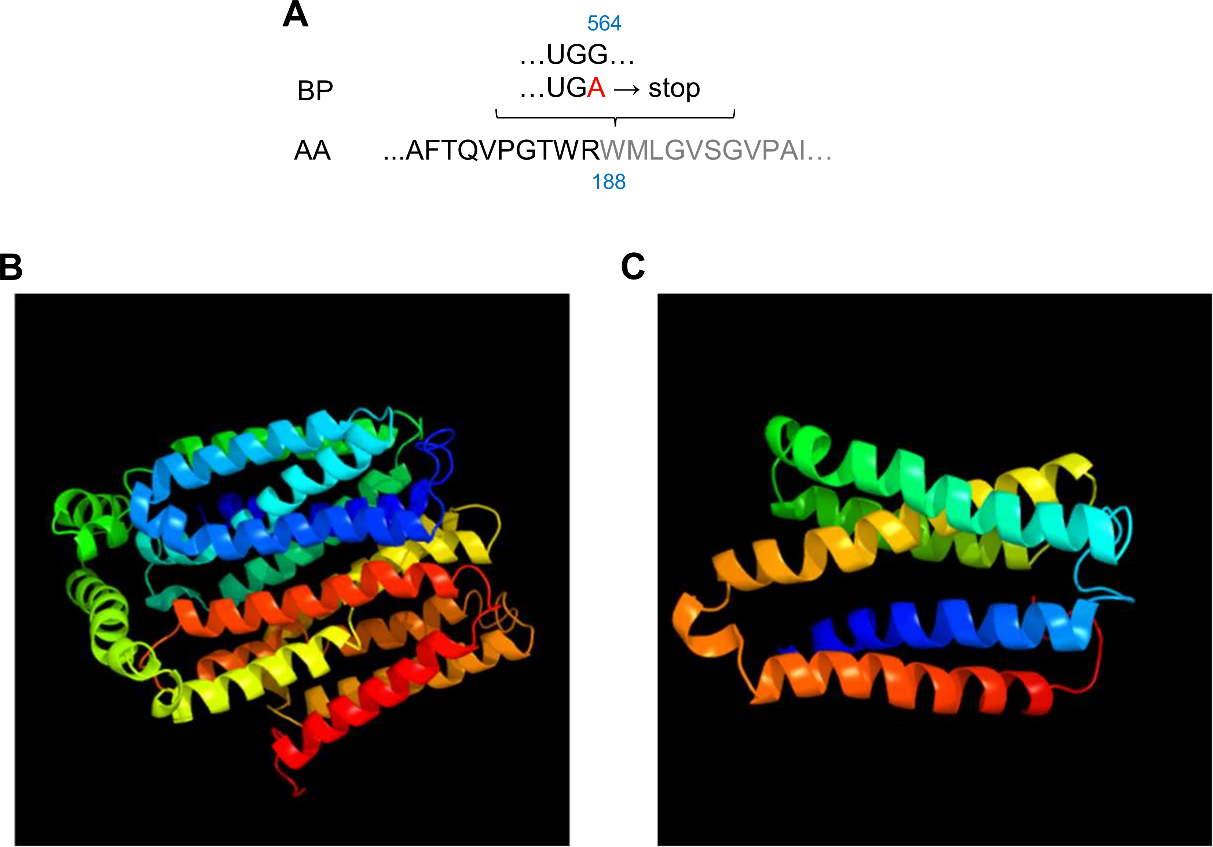


**Supplemental Figure 4: Characterization of *bvint1;1* mutants.**

**A)** Plants with a mutation in the *BvINT1;1* coding sequence were identified in an EMS-treated sugar beet population; the mutation consisted of a conversion of a guanine to adenine at position 564. Corresponding base exchange leads to an early stop at amino acid position 188 in the sequence of *Bv*INT1;1. Phyre2 structure prediction of **B)** functional *Bv*INT1;1 and **C)** truncated *Bv*INT1;1 reveal the formation of 12 or five α-helices respectively. Helices are colored according to the colors of a rainbow from N- to C- terminus. For functional *Bv*INT1;1 prediction 420 residues (84% of the query sequence) were modeled with 100% confidence by the single highest-scoring template, while for truncated *Bv*INT1;1 prediction 154 residues (82% of the query sequence) were modeled with 99.8% confidence by the single highest-scoring template.


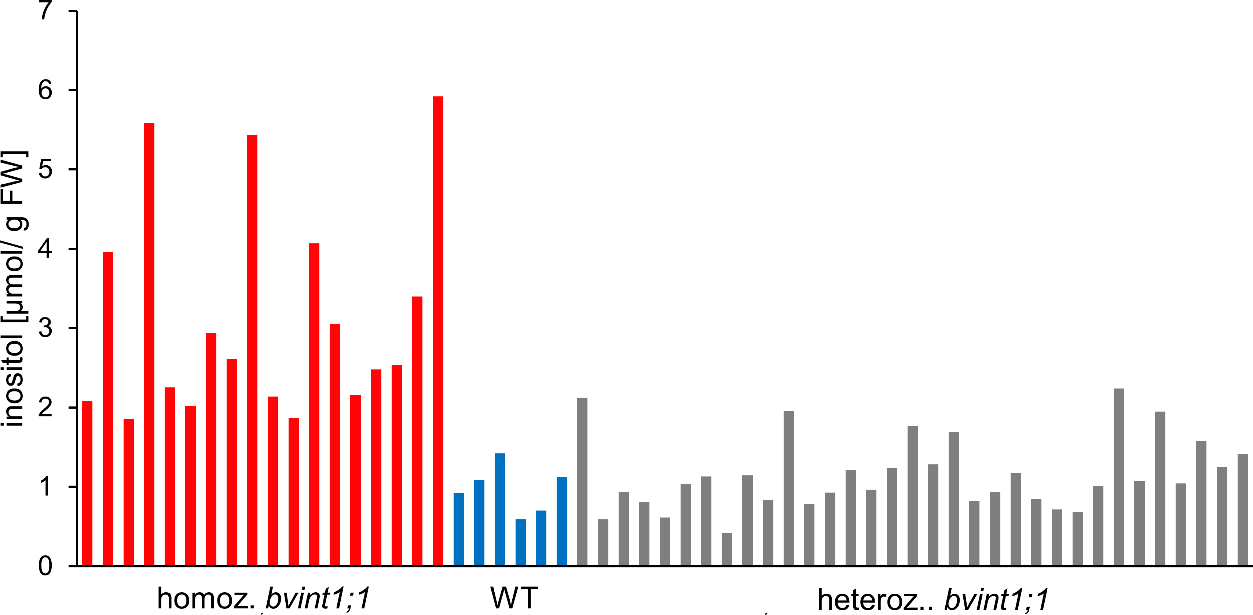


**Supplemental Figure 5: Inositol content in leaves of homozygous and heterozygous *bvint1;1* plants and wild types.**

Inositol contents were measured in µmol/ g FW of shoot material from 18 individual homozygous *bvint1;1* plants, six wild type plants, and 33 heterozygous *bvint1;1* sugar beets.


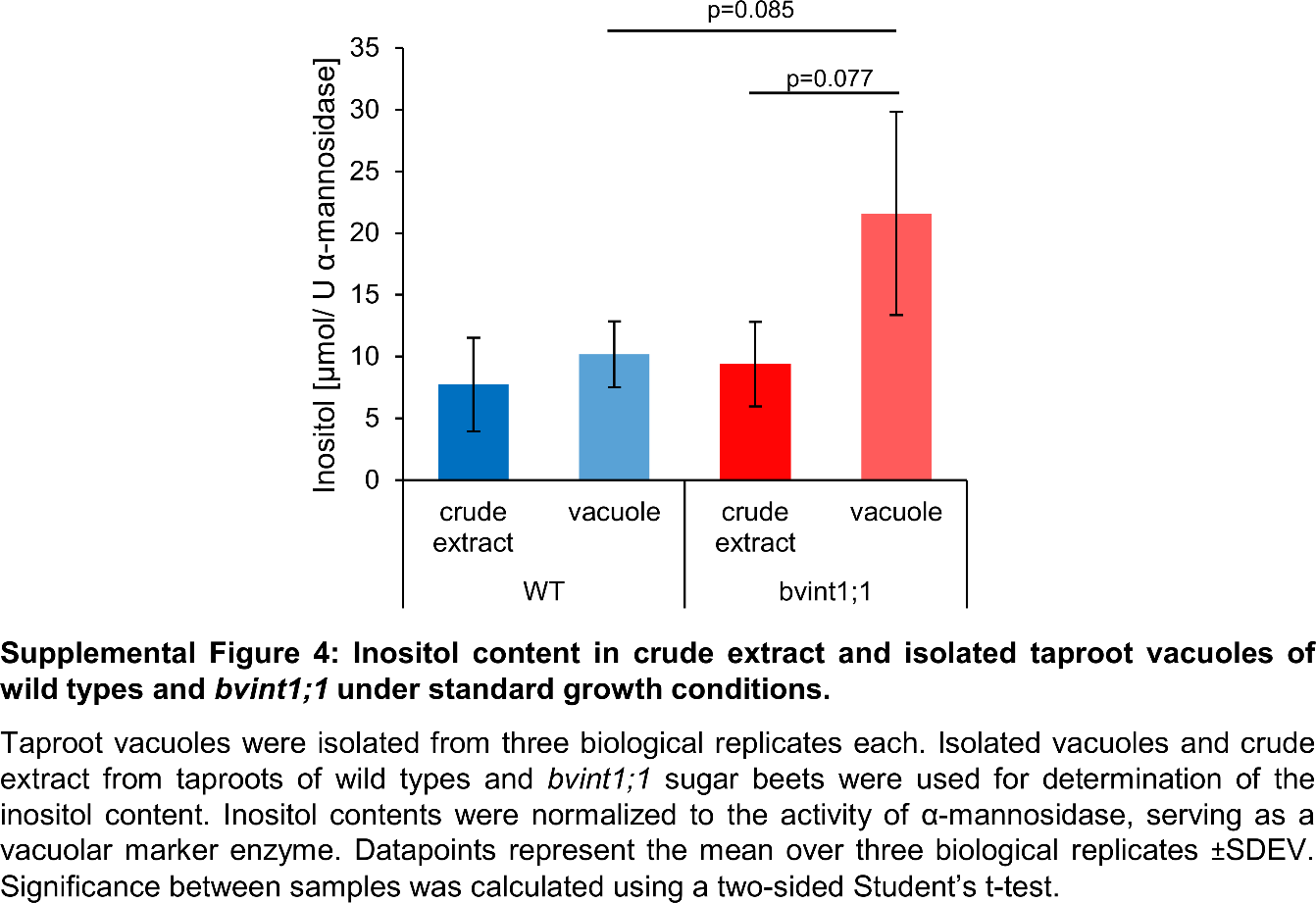


**Supplemental Figure 6: Inositol content in crude extract and isolated taproot vacuoles of wild types and *bvint1;1* under standard growth conditions.**

Taproot vacuoles were isolated from three biological replicates each. Isolated vacuoles and crude extract from taproots of wild types and *bvint1;1* sugar beets were used for determination of the inositol content. Inositol contents were normalized to the activity of α-mannosidase, serving as a vacuolar marker enzyme. Datapoints represent the mean over three biological replicates ±SDEV. Significance between samples was calculated using a two-sided Student’s t-test.


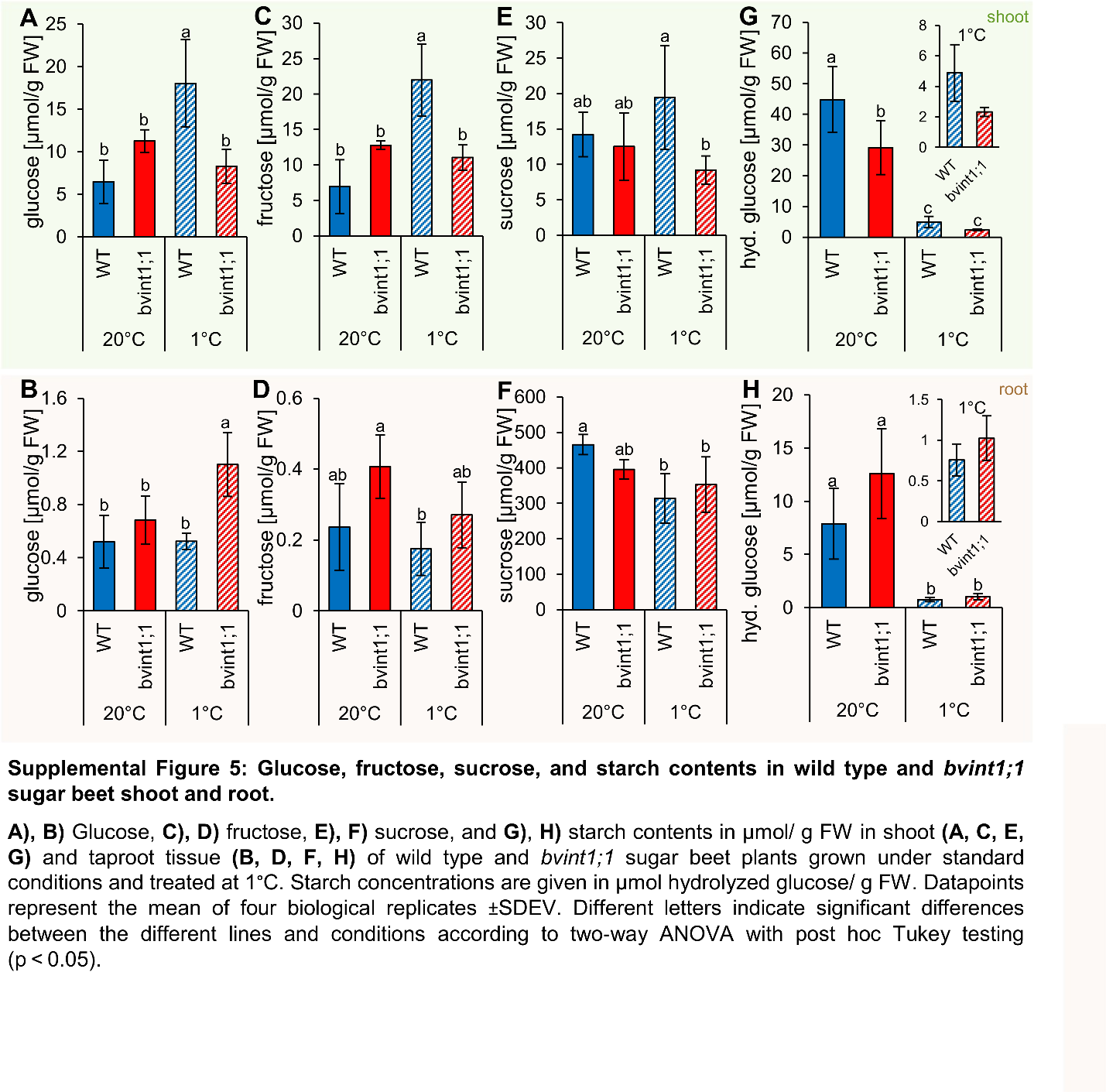


**Supplemental Figure 7: Glucose, fructose, sucrose, and starch contents in wild type and *bvint1;1* sugar beet shoot and root.**

**A), B)** Glucose, **C), D)** fructose, **E), F)** sucrose, and **G)**, **H)** starch contents in µmol/ g FW in shoot **(A, C, E, G)** and taproot tissue **(B, D, F, H)** of wild type and *bvint1;1* sugar beet plants grown under standard conditions and treated at 1°C. Starch concentrations are given in µmol hydrolyzed glucose/ g FW. Datapoints represent the mean of four biological replicates ±SDEV. Different letters indicate significant differences between the different lines and conditions according to two‐way ANOVA with post hoc Tukey testing (p < 0.05).
