# Supplementary material for: The Vacuolar Inositol Transporter BvINT1;1 Contributes to Raffinose Biosynthesis and Reactive Oxygen Species Scavenging During Cold Stress in Sugar Beet
Source: Plant Cell Environ. 2025 Jan 8;48(5):3471–86. doi: 10.1111/pce.15367 (PMC11963481; doi:10.1111/pce.15367)
Supplement: Supplementary file 2 — Supporting information. [file PCE-48-3471-s001.docx]

**Supplemental Tables S1-S3**

**The vacuolar inositol transporter *Bv*INT1;1 contributes to raffinose biosynthesis and reactive oxygen species scavenging during cold stress in sugar beet**

Johannes Berg^1^, Cristina Martins Rodrigues^1^, Claire Scheid^1^, Yana Pirrotte^1^, Cristiana Picco^2^, Joachim Scholz-Starke^2^, Wolfgang Zierer^3^, Olaf Czarnecki^4^, Dieter Hackenberg^4^, Frank Ludewig^4^, Wolfgang Koch^4^, H. Ekkehard Neuhaus^1^, Christina Müdsam^3^, Benjamin Pommerrenig^1§^, Isabel Keller^1*^

**Table S1:** Primers used in this study.

**Table S2:** Expression of enzymes involved in inositol and raffinose family oligosaccharides synthesis in wild type and *bvint1;1* sugar beet shoot and root.

**Table S3:** Expression of ROS-Marker Enzymes in wild type and *bvint1;1* sugar beet shoot and root.

**Supplemental Table S1: Primers used in this study.**

| **primer** | **sequence (5´- 3´)** | **purpose** | **reference** |
| --- | --- | --- | --- |
| *Bv*INT1;1-5'BspHI-f | TCATGACACTTGATTCAATTCCAGGGAGT | cloning subcellular localization | this work |
| *Bv*INT1;1-3'BspHl-r | TCATGACGGCACGCTGATTTTCG |  |  |
| *Bv*INT1.2-5’NcolCC-f | ССАTGGССАTGAСTTTGGАTTСААТ |  |  |
| *Bv*INT1.2-3'ATGG-r | CCATGGACTTGTСTTСАСCATTATTTAT |  |  |
| *Bv*INT2-5‘TCATG-f | TCATGATGGTGGAAGGTGGAGTA |  |  |
| *Bv*INT2-3‘GBspHl-r | ТСАTGACAGСTTСАСTAATTTTGTTCTC |  |  |
| *Bv*INT1;1-LL/AA-X-r | TTTCGAACCCATTCCCAGAATCAGCAGCGC |  |  |
| pNBI16/22-*Bv*lNT1.1-f | CGGGGCTGAGGCTTAATATGACACTTGATTCAATTCCAGGCA | cloning oocyte expression |  |
| pNBI21-*Bv*INT1;1-f | GTGGGCTGAGGCTTAATATGACACTTGATTCAATTCCAGGGA |  |  |
| *Bv*INT1;1-pNBI16/21-r | ATTCGCTGAGGTTTAGGCACGCTGATTTTCCAACCCATT |  |  |
| *Bv*INT1;1-pNBI22-r | CCAGGCTGAGGTTTAAGGCACGCTGATTTTCGAACCCATT |  |  |
| UBC9-fw | AAGGAGCAGTGGAGTCCTGC | RT-qPCR |  |
| UBC9-rev | TGTGTCCAGCTCCTTGCGG |  |  |
| GOLS1-fw | TTGGTGAAGAAGTGGTGGGA |  | Keller et al., 2021a |
| GOLS1-rev | AGCAGCAGAAGGAGCAGTAA |  |  |
| GOLS2-fw | CTGAGGACAAGTTAGGCCCA |  |  |
| GOLS2-rev | AATGTTTTCAGGGTGACGCC |  |  |
| GOLS3-fw | CGCATTTGGGAGTTTGTGGA |  |  |
| GOLS3-rev | GCCAGCACACTCTATTTGGG |  |  |
| RS2-fw | GCAGCATCTCATTTACGCCA |  |  |
| RS2-rev | AAACCGGCCACATCCTCTTA |  |  |
| RS5-fw | AGCCATTCCCCATCAAAGGA |  |  |
| RS5-rev | CAAATGCGATCGACTCCCAG |  |  |
| ZAT10-fw | AACATACAAGTGCGGCGTTT |  |  |
| ZAT10-rev | GCAGATTGAGCAGACGTGAG |  |  |
| ZAT12-fw | GCCAATTTGCCTCCTTCCAA |  |  |
| ZAT12-rev | TATGACCTCCCAACGCTTGT |  |  |
| SOD-fw | ATTCTCGCTTCCCACCCTAC |  |  |
| SOD-rev | AATGGTGAGGGGTTTAGGGG |  |  |
| CAT-fw | GGCTGGCAAAGTACACTACG |  |  |
| CAT-rev | TCCTCAGGCCATGTCTTTGT |  |  |
| APX-fw | CGAGAAGGCCAAGAGAAAGC |  |  |
| APX-rev | GGCTCCAACAACCTAACAGC |  |  |
| MDAR-fw | AAAACTGTCGTGGTTGGTGG |  |  |
| MDAR-rev | CCGTCACCTCTCCGTTATCA |  |  |
| DHAR-fw | CCTTTAAACGTCCGGTGGTC |  |  |
| DHAR-rev | AGCTTGTTCGGAGTGGTGAT |  |  |
| GPX-fw | CACAGTGTGGGTTGACATCA |  |  |
| GPX-rev | GGAGCTGTGTTTGAACCGTT |  |  |
| GR-fw | AGGGCTGTTGTCGCTAGAAA |  |  |
| GR-rev | GTTCAACACCAACAGCGTC |  |  |
| AOX1α-fw | tggttggagggatgcttctt |  | this work |
| AOX1α-rev | aagaagactccctggacagc |  |  |
| UCP1-fw | ttgctgcacttacaactggc |  |  |
| UCP1-rev | ggccttaagtccttcctgct |  |  |

**Supplemental Table 2: Expression of enzymes involved in inositol and raffinose family oligosaccharides synthesis in wild type and *bvint1;1* sugar beet shoot and root.**

Calculated fold change ± standard error of gene expression in wild type plants grown at 1°C and *bvint1;1* plants grown at 20°C and 1°C in comparison to the wild type under ambient conditions. The calculated fold changes form the basis of the heat map representation shown in Figure 4G.

|  |  | 20°C | | 1°C | |
| --- | --- | --- | --- | --- | --- |
|  |  | WT | *bvint1;1* | WT | *bvint1;1* |
| *GOLS1* | shoot | 1.00 ± 0.08 | 1.41 ± 0.10 | 27.53 ± 5.70 | 40.53 ± 11.98 |
|  | root | 1.00 ± 0.25 | 1.05 ± 0.28 | 56.64 ± 8.49 | 132.26 ± 22.86 |
| *GOLS2* | shoot | 1.00 ± 0.10 | 1.87 ± 0.07 | 4.75 ± 0.42 | 19.50 ± 3.64 |
|  | root | 1.00 ± 0.78 | 0.10 ± 0.02 | 0.57 ± 0.15 | 17.49 ± 1.28 |
| *GOLS3* | shoot | nd | nd | nd | nd |
|  | root | 1.00 ± 0.60 | 7.31 ± 3.42 | 118572.33 ± 33254.74 | 1024497.6970 ± 304558.45 |
| *RS2* | shoot | 1.00 ± 0.13 | 0.41 ± 0.05 | 2.21 ± 0.60 | 0.93 ± 0.06 |
|  | root | 1.00 ± 0.18 | 2.53 ± 0.40 | 29.07 ±4.63 | 66.51 ± 7.18 |
| *RS5* | shoot | 1.00 ± 0.14 | 0.50 ± 0.05 | 0.43 ± 0.08 | 0.68 ± 0.10 |
|  | root | 1.00 ± 0.20 | 1.93 ± 0.31 | 87.95 ± 11.64 | 262.82 ± 25.04 |

**Supplemental Table 3: Expression of ROS-Marker Enzymes in wild type and *bvint1;1* sugar beet shoot and root.**

Calculated fold change ± standard error of gene expression in wild type plants grown at 1°C and *bvint1;1* plants grown at 20°C and 1°C in comparison to the wild type under ambient conditions. The calculated fold changes form the basis of the heat map representation shown in Figure 5.

| gene symbol | tissue | 20°C | | 1°C | |
| --- | --- | --- | --- | --- | --- |
|  |  | WT | *bvint1;1* | WT | *bvint1;1* |
| *ZAT10* | shoot | 1.00 ± 0.09 | 0.55 ± 0.05 | 1.31 ± 0.08 | 2.64 ± 1.01 |
|  | root | 1.00 ± 0.31 | 1.51 ± 0.28 | 15.92 ± 0.67 | 34.92 ± 0.65 |
| *ZAT12* | shoot | 1.00 ± 0.04 | 0.65 ± 0.06 | 0.62 ± 0.17 | 0.44 ± 0.11 |
|  | root | 1.00 ± 0.17 | 0.49 ± 0.05 | 2.35 ± 0.33 | 5.26 ± 1.15 |
| *AOX1a* | shoot | 1.00 ± 0.23 | 0.64 ± 0.06 | 0.29 ± 0.02 | 0.26 ± 0.05 |
|  | root | 1.00 ± 0.15 | 6.91 ± 1.17 | 12.37 ± 4.16 | 48.62 ± 7.99 |
| *UCP1* | shoot | 1.00 ± 0.12 | 0.49 ± 0.07 | 3.36 ± 0.07 | 2.48 ± 0.24 |
|  | root | 1.00 ± 0.21 | 0.44 ± 0.03 | 1.63 ± 0.12 | 1.83 ± 0.05 |
| *SOD* | shoot | 1.00 ± 0.16 | 8.23 ± 0.74 | 0.72 ± 0.10 | 2.43 ± 0.26 |
|  | root | 1.00 ± 0.13 | 2.61 ± 0.45 | 2.12 ± 0.37 | 16.46 ± 2.72 |
| *CAT* | shoot | 1.00 ± 0.23 | 0.61 ± 0.01 | 0.38 ± 0.04 | 0.32 ± 0.05 |
|  | root | 1.00 ± 0.11 | 1.17 ± 0.18 | 3.82 ± 0.37 | 6.97 ± 0.25 |
| *APX* | shoot | 1.00 ± 0.10 | 3.73 ± 0.49 | 1.51 ± 0.1 | 4.33 ± 0.68 |
|  | root | 1.00 ± 0.13 | 1.38 ± 0.14 | 2.07 ± 0.34 | 4.10 ± 0.66 |
| *MDAR* | shoot | 1.00 ± 0.05 | 0.57 ± 0.08 | 0.62 ± 0.08 | 0.42 ± 0.09 |
|  | root | 1.00 ± 0.22 | 4.28 ± 0.21 | 4.07 ± 1.46 | 6.75 ± 0.39 |
| *DHAR* | shoot | 1.00 ± 0.07 | 0.54 ± 0.08 | 1.11 ± 0.13 | 0.78 ± 0.15 |
|  | root | 1.00 ± 0.17 | 0.57 ± 0.09 | 3.54 ± 0.49 | 3.08 ± 0.17 |
| *GPX* | shoot | 1.00 ± 0.17 | 0.70 ± 0.15 | 0.52 ± 0.12 | 0.31 ± 0.04 |
|  | root | 1.00 ± 0.04 | 0.99 ± 0.12 | 0.88 ± 0.20 | 0.41 ± 0.05 |
| *GR* | shoot | 1.00 ± 0.49 | 478.85 ± 101.67 | 1.00 ± 0.29 | 129.54 ± 19.53 |
|  | root | 1.00 ± 0.24 | 5.02 ± 1.43 | 5.43 ± 0.70 | 56.56 ± 5.13 |
